# Structure-Based Design and In-Silico Evaluation of Computationally Proposed Curcumin Derivatives as Potential Inhibitors of the Coronaviral PLpro Enzymes

**DOI:** 10.3390/ph18060798

**Published:** 2025-05-26

**Authors:** Hakan Alici

**Affiliations:** Department of Physics, Faculty of Science, Zonguldak Bülent Ecevit University, 67100 Zonguldak, Türkiye; hakanalici@beun.edu.tr

**Keywords:** curcumin derivative, coronavirus, papain-like protease, molecular docking, molecular dynamics, MM/PBSA analysis, ADMET property, antiviral agent, bioavailability

## Abstract

**Background/Objectives:** Highly pathogenic coronaviruses (CoVs), including SARS-CoV, MERS-CoV, and SARS-CoV-2, continue to pose a significant threat to global public health. Therefore, this situation highlights the urgent need for effective broad-spectrum antiviral agents. Curcumin, a naturally occurring polyphenol known for its antiviral and anti-inflammatory properties, faces limitations such as poor bioavailability and rapid metabolic degradation, restricting its practical therapeutic application. **Methods:** To address these limitations, this study introduces a novel design strategy aimed at 42 new curcumin derivatives with improved pharmacokinetic profiles, specifically targeting the conserved coronavirus enzyme papain-like protease (PLpro). A comprehensive in silico evaluation was performed, including ADMET (Absorption, Distribution, Metabolism, Elimination, and Toxicity) analysis, molecular docking, molecular dynamics (MD) simulations, and Molecular Mechanics/Poisson-Boltzmann Surface Area (MM/PBSA) calculations. **Results:** Extensive pharmacokinetic and toxicological assessments (ADMET analyses) identified 19 derivatives exhibiting optimal drug-like characteristics according to Lipinski’s Rule of Five (Ro5). Molecular docking analyses demonstrated that these novel derivatives possess significantly enhanced binding affinities to PLpro enzymes from SARS-CoV, MERS-CoV, and SARS-CoV-2 compared to standard antiviral agents and natural curcumin. Further validation through MD simulations and MM/PBSA calculations confirmed the structural stability and robust interactions of the most promising derivatives within the SARS-CoV PLpro active site. **Conclusions:** The results of this study provide essential structural and functional insights, reinforcing the potential of these newly developed curcumin derivatives as potent, broad-spectrum antiviral agents effective against current and future coronavirus threats.

## 1. Introduction

CoVs are a large family of RNA viruses that can cause diseases ranging from mild respiratory tract infections to severe diseases such as Severe Acute Respiratory Syndrome (SARS), Middle East Respiratory Syndrome (MERS), and Coronavirus Disease 2019 (COVID-19) [1,2]. As it is known, highly pathogenic CoVs such as SARS-CoV, MERS-CoV, and SARS-CoV-2 pose serious public health threats globally due to their rapid spread and high mortality rates [3,4,5]. Furthermore, the high evolutionary adaptability of CoVs and their ability to generate new variants significantly elevate the risk of future pandemics [6,7,8,9]. Considering the limited efficacy, potential resistance development, and undesirable side effects of existing antiviral agents, there is an urgent need for the development of novel broad-spectrum antivirals. Therefore, the rational design of broad-spectrum antiviral agents is a critical research area in the fight against viral diseases [10,11].

At this point, PLpro, which plays a significant role in the life cycle of CoVs, is a highly conserved enzyme in all CoVs and has important functions in both viral replication and immune system escape processes [12,13,14]. Here, it is well established that the primary function of PLpro is to activate large viral polyproteins required for viral replication by cleaving them into functional subunits [15,16]. The second function is to suppress the host immune response, and in this context, PLpro helps the virus to escape from the immune system by removing modifications of ubiquitin (Ub) and interferon-stimulated gene 15 (ISG15) proteins [14,17,18,19]. Thanks to this bidirectional function and highly conserved structure, PLpro is considered one of the priority targets in broad-spectrum antiviral drug design [14].

In our previous study [20], the activities of various curcumin derivatives on the main enzyme structures of the SARS-CoV-2 virus were systematically evaluated by computational methods, and as a result, it was reported that the proposed new curcumin derivatives may be especially more effective on the PLpro of SARS-CoV-2. With this in mind, in the present study, we introduce a set of 42 synthetically accessible novel curcumin derivatives specifically designed to inhibit PLpro enzymes from three major CoVs.

As it is known, curcumin is a natural polyphenol with known antiviral and anti-inflammatory properties and has been investigated for a long time due to its therapeutic potential [21,22]. However, its low bioavailability and rapid metabolism are the main factors limiting its pharmacological activity [23,24,25]. To overcome these limitations, various approaches such as chemical modifications and formulation with carrier systems have been extensively explored in the literature [26,27,28,29,30]. In addition to its broad-spectrum biological activities, curcumin’s chemically tractable scaffold and favorable safety make it a highly suitable platform for rational structure-based drug design. In this study, curcumin was not merely selected for its intrinsic antiviral properties but also as a modular and modifiable pharmacophore that enables the generation of optimized analogs specifically tailored to inhibit the conserved PLpro enzyme in CoVs.

However, most existing curcumin-based antiviral studies have focused primarily on SARS-CoV-2, often neglecting the need for inhibitors effective against a broader range of CoVs, such as SARS-CoV and MERS-CoV [31,32,33]. Also, a significant proportion of prior research has centered on viral spike proteins and main proteases (3CLpro) and RNA-dependent RNA polymerase (RdRP), leaving the PLpro—a key enzyme in viral replication and immune evasion—relatively underexplored [14,20]. In light of this gap, the current study advances the field by introducing a set of synthetically accessible curcumin derivatives specifically designed to inhibit PLpro enzymes from three major pathogenic CoVs. This approach not only addresses a critical viral target but also responds to the growing need for structurally optimized curcumin analogs that demonstrate efficacy across multiple coronavirus strains. Furthermore, the key innovative aspect of this study is the design of new curcumin derivatives that will both be synthesizable in laboratory conditions and exhibit improved pharmacokinetic and pharmacological profiles. A synthetic pathway was also proposed to assess the practical feasibility of synthesizing the designed curcumin derivatives, and thus it provides a clear roadmap for experimental research groups.

In recent years, several novel antiviral agents have been developed and investigated for their efficacy against SARS-CoV-2 and its variants, including the highly transmissible Omicron [34]. Among these, nucleoside and nucleotide analogs such as molnupiravir, azvudine, remdesivir, and didanosine have shown promise by targeting RdRp, while ensitrelvir, simnotrelvir, and teriflunomide function primarily as 3CLpro inhibitors [35,36,37,38,39,40,41,42,43,44,45]. In parallel, recent computational studies using molecular docking and QSAR modeling have also proposed repurposed drug candidates and predictive descriptor-based screening strategies for 3CLpro inhibition [46,47]. Recent studies have also highlighted agents like Taroxaz-104 and Cyanorona-20, which exhibit dual inhibitory effects and favorable pharmacokinetics, further supporting the development of novel RdRp-targeting analogs [45,48]. In addition to synthetic antivirals, naturally derived compounds like cordycepin, riboprine, and phytochemicals from sources such as ginger, pineapple, and strychnine bush have been explored for their potential inhibition of viral enzymes, including PLpro and 3CLpro [38,39,40,41,49,50]. Notably, compounds such as ananas-26 and zingiberenol have demonstrated superior binding affinities to 3CLpro compared to benchmark antivirals like remdesivir [50]. Moreover, chemical classes such as quinolines, isoquinolines, gallates, and oxadiazoles have been recognized for their broad-spectrum antiviral activities [37,42,43,44,49]. For example, oxadiazole-based molecules such as CoViTris2020 and ChloViD2020 have shown multitarget inhibition of PLpro, 3CLpro, and RdRp enzymes in SARS-CoV-2, with high potency across variants [49]. Furthermore, many studies have employed computational methods to evaluate binding affinity, ADMET properties, and drug-likeness, highlighting gaps in the optimization of synthetic pathways and the stability of these antiviral candidates [9,10,11,12].

Considering the limited exploration of PLpro compared to other viral targets and the scarcity of multitarget compounds active across multiple coronavirus variants, our current study builds upon this foundation by introducing a new series of curcumin derivatives aimed at PLpro inhibition, supported by rigorous computational evaluation and pharmacokinetic profiling.

Moreover, as it is known, it is of great importance to examine the pharmacokinetic and toxicological profiles of compounds in order to evaluate them as potential drug candidates at the design stage of a candidate [51,52,53,54,55,56,57]. In this context, ADMET analyses were first performed, and curcumin derivatives with pharmacologically favorable properties were selected according to certain filtering criteria. This filtering process was conducted based on Ro5 rules, a widely accepted criterion for drug-likeness [58,59]. After this stage, only compounds with high bioavailability, low toxicity risk, and strong interaction with PLpro were subjected to advanced computational analyses.

Given these significant considerations, this study aims to evaluate the inhibitory potential of selected curcumin derivatives against PLpro enzymes isolated from different coronavirus species such as SARS-CoV, MERS-CoV, and SARS-CoV-2 using computational methods. The efficacy of these curcumin derivatives was first assessed via Molecular Docking methods. Subsequently, molecular dynamics (MD) simulations were utilized to analyze their interactions with PLpro regarding binding stability, key interactions, and conformational dynamics. The obtained findings reveal critical structural and dynamic information about the inhibitory effects of new curcumin derivatives on PLpro. Additionally, it is aimed that insights from this study will contribute to the rational design of next-generation broad-spectrum antiviral inhibitors and inform potential therapeutic strategies against current and future coronavirus threats.

## 2. Results and Discussion

### 2.1. Design Strategy and Structural Modifications

Although curcumin is a natural polyphenol with a wide range of biological activities, its pharmacological use is restricted due to its low bioavailability, rapid metabolism, and limited cellular penetration [60,61]. In order to overcome these problems, there are various studies on curcumin derivatives in the literature [62,63]. At the same time, in our previous study, we applied various structural modifications to curcumin derivatives and evaluated the binding affinities of these derivatives to SARS-CoV2 main enzymes and demonstrated their activities [20].

In the current study, a new design strategy was adopted for the optimization of the central skeleton and side groups, different from previously proposed curcumin derivatives. In this context, the central skeleton structure of the newly designed compounds has a dense aromatic ring system that offers a more rigid and planar structure, unlike the classical curcumin skeleton. The two carbonyl (C=O) groups in the center of curcumin undergo keto-enol tautomerism, resulting in structural flexibility, and this flexibility has the potential to create conformational uncertainty when settling into the binding site [64,65]. At this point, it was aimed to eliminate these conformational ambiguities caused by the β-diketone system of the central skeleton by modification with aromatic rings containing two hydroxyl (-OH) and two methoxy (-OCH_3_) groups. Thus, by adding a more rigid core structure, it was aimed to provide stronger binding of curcumin ligands to the protein. The two carbonyl (C=O) groups in the middle of curcumin undergo keto-enol tautomerism, causing structural flexibility, and this flexibility has the potential to create conformational uncertainty when positioned in the binding site. At this point, it was aimed to eliminate these conformational uncertainties caused by the β-diketone system of the central skeleton by modification with aromatic rings containing two hydroxyl (-OH) and two methoxy (-OCH_3_) groups [66]. Thus, by adding a more rigid core structure, it was aimed to provide stronger binding of curcumin ligands to the protein. Here, this design strategy also aligns with the emerging field of skeletal editing (SKED), which focuses on atom-level modifications of molecular cores to enhance pharmacokinetics and optimize therapeutic efficacy, as recently discussed in medicinal chemistry literature [67].

**Figure 1:**
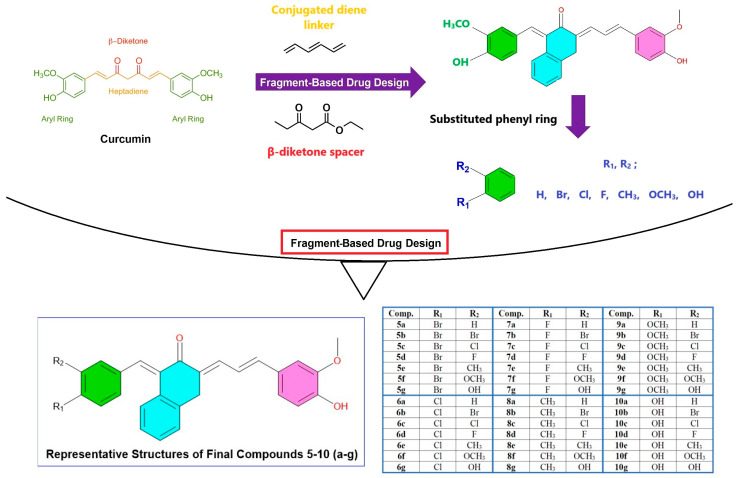
General structures of the final target compounds (**5–10(a–g)**) with variable substituents R_1_ and R_2_ as detailed in the accompanying table.

In the design of side groups, various modifications were introduced by considering the hydrophobic–hydrophilic balance, electrostatic interactions, and specific interaction sites within the binding pocket. As in our previous study, halogen (F, Cl, Br), methoxy (-OCH_3_), and hydroxyl (-OH) groups were strategically added [20]. However, in the presented study, the positioning of the side groups was made more systematic, allowing for a wider range of variability in both R_1_ and R_2_ positions. At this point, it is well established that halogen-containing compounds have the potential to strengthen the electrostatic interactions in the PLpro binding pocket. Especially derivatives containing Fluorine (F) can stabilize the position of the ligand by interacting with hydrogen bond donors in the binding pocket, while Chlorine (Cl) and Bromine (Br)-containing ones can show stronger binding affinities by increasing van der Waals interactions. On the other hand, it is known that compounds containing methoxy (-OCH_3_) groups increase binding stability and improve solubility and pharmacokinetic profile. Also, derivatives with added methoxy groups are expected to facilitate cellular uptake by increasing solubility in the binding site as well as hydrophobic and aromatic interactions. In addition, compounds containing hydroxyl (-OH) groups can increase binding stability by providing direct interaction with the hydrogen bond network in the active site of PLpro. Considering all this, this new design strategy aims to increase both inhibitory activity and pharmacokinetic parameters by determining functional groups that will establish stronger interactions with the binding site of PLpro, unlike previous curcumin derivatives. Moreover, our main starting point here is the easy experimental applicability of the proposed modifications, and in this context, the new curcumin derivatives and their synthesis scheme proposed to guide experimental groups are exhibited in Scheme S1. As can be seen from the synthesis scheme in this study, 42 new curcumin derivatives are proposed. Also, Figure 1 presents the Fragment-Based Drug-Design (FBDD) scheme, which enables the reader to easily trace the general structures of the final target compounds. Furthermore, predicted spectral data (^1^H-NMR and ^13^C-NMR) for selected compounds were generated using cheminformatics tools and are included in the Supplementary Information (Figures S2–S7) for potential future experimental validation. Also, general structures of the final target compounds.

### 2.2. ADMET Evolation

#### 2.2.1. Evaluation of Drug-Likeness and Pharmacokinetic Properties of Curcumin Derivatives Based on Ro5

Understanding the pharmacokinetic and toxicological profiles of drug candidates is of great importance for both their efficacy and safety [59,68]. At this point, after a compound is taken into the body, the absorption, distribution, metabolism, and excretion (ADME) process, along with the compound’s toxic potential, are among the determining critical factors in the clinical development process [69,70]. In this context, inadequate study or evaluation of ADMET parameters causes many biologically active compounds to fail before they reach clinical applications. Therefore, the pharmacokinetic profiles of the compounds designed in this study were evaluated in detail. For this purpose, in order to put our study on a solid basis, the proposed compounds were exploited according to Ro5 rules to determine whether they exhibit drug-likeness properties, and they were subsequently subjected to filtering in this context [59]. Ro5 rules are one of the main filtering approaches commonly used to estimate the ability of compounds to pass through cell membranes by passive diffusion and hence their oral bioavailability, and they also allow for the discrimination between drug-like and non-drug-like molecules [68,71]. According to these rules, a drug-like compound must fulfill the following criteria [59]:

- Molecular Weight (MW) ≤ 500 Da
- Hydrogen Bond Donor Number (HBD) ≤ 5
- Hydrogen Bond Acceptor Number (HBA) ≤ 10
- Lipophilicity (logP) ≤ 5

In this respect the compliance of the proposed curcumin derivatives, curcumin, and selected reference pharmaceuticals with Ro5 criteria, along with their associated pharmacokinetic properties, is listed in Table 1. In this table, MW, HBA, and HBD represent the physicochemical parameters of the compounds, while logP is a critical parameter that determines the lipophilicity of a compound and its potential for cellular uptake via passive membrane diffusion [72,73].

When the table is analyzed, it is seen that the most frequently violated parameters of the Ro5 rules for the proposed curcumin derivatives are MW and lipophilicity. Especially compounds **5b**, **5c**, **5f**, **6b**, and **9b** do not meet the Ro5 criteria since they violate both criteria by exceeding the limit values in terms of both MW and logP. Also, compounds **5a**, **5d**, **5e**, **6a**, **6c**, **6d**, **6e**, **6f**, **7b**, **7c**, **7e**, **8a**, **8b**, **8c**, **8d**, **8e**, **8f**, and **9c** violate Ro5 rules 1 time only because the logP value is greater than 5. On the other hand, it is observed that compounds **7a**, **7d**, **7f**, **7g**, **8g**, **9a**, **9d**, **9e**, **9f**, **9g**, **10a**, **10b**, **10c**, **10d**, **10e**, **10f**, and **10g** meet the Ro5 criteria completely, and hence they exhibit drug-likeness properties. Although, according to Lipinski’s rule, it is generally acceptable for an orally active drug to violate at most one of the Ro5 criteria [59], we eliminated compounds that violated at least one criterion from the 42 compounds designed to provide a solid basis for our study, and a total of 19 compounds were included in the advanced pharmacokinetic analyses.

In addition, curcumin, which is our starting point in the study, has important biological activities in terms of pharmacokinetics; however, as seen in the table, its logP value was determined as 1.47, and therefore it is limited in terms of lipophilicity. On the other hand, the majority of the curcumin derivatives evaluated in the study exhibit a balanced lipophilicity profile with logP values between 4 and 5. This suggests that the proposed new curcumin derivatives may offer improved bioavailability compared to curcumin. In addition, the filtered derivatives showed better Ro5 compatibility compared to the reference drugs Lopinavir and Remdesivir. Accordingly, considering that Lopinavir has one Ro5 violation and Remdesivir has two Ro5 violations, it can be predicted that the compounds in the study may be pharmacokinetically more advantageous than the existing reference drugs.

In summary, considering the pharmacokinetic suitability of the compounds selected in the study, their effective passage through the cell membrane, their optimal lipophilicity levels, and their comparative advantages with reference drugs, it can be said that they have the potential to be evaluated as drug candidates with high oral bioavailability. In this context, it was decided to subject the 19 compounds selected by filtering in accordance with Ro5 rules to further analyses for detailed examination of ADMET profiles.

#### 2.2.2. Absorption and Oral Bioavailability

The therapeutic efficacy of a drug is directly related to its bioavailability, and one of the most important determinants of this bioavailability is the absorption process. Compounds that can be absorbed sufficiently from the intestines can pass into the systemic circulation more efficiently and thus show the desired pharmacological effect in target tissues [74]. Therefore, the evaluation of the gastrointestinal absorption capacity of drug candidates is a critical stage in the pharmaceutical development process [75]. Accordingly, the calculated absorption properties of the compounds are listed in Table 2.

When the table is examined, the intestinal absorption rate of curcumin was determined as 82.19%. On the other hand, all of the compounds in the study showed absorption above 89%, and this rate reached up to 97% in some compounds. The compounds with the highest absorption rates were **7f** (97.097%), **9e** (96.177%), and **7d** (96.11%), respectively. In general, these high absorption rates indicate that the proposed curcumin derivatives can be administered orally, and their passage into the systemic circulation can be much more efficient than curcumin.

Also, significant differences are observed in terms of Caco-2 permeability (log Papp), another parameter that determines intestinal permeability [76,77]. At that point, it can be said that the low intestinal permeability of curcumin, determined as −0.093 as seen in the table, is one of the main factors limiting its access to the systemic circulation. In contrast, among the compounds suggested in the study, there are compounds with high intestinal permeability, such as **7d** (1.296), **7f** (1.258), and **9d** (1.259). These results indicate that compared to curcumin, the proposed derivatives in the study are absorbed more efficiently and can pass into the systemic circulation more easily.

In addition, interaction with P-glycoprotein (P-gp) is an important factor in absorption processes. P-gp is a carrier protein found in intestinal epithelial cells and can limit systemic absorption of compounds that are its substrates by expelling them from the cell [78,79]. Curcumin and all compounds in the study except **7d** and **7f** are P-gp substrates, which may be a factor that reduces bioavailability. However, the proposed derivatives in the study are also P-gp inhibitors, and this situation suggests that they have the potential to increase their own absorption by suppressing the activity of this carrier protein. In particular, compounds **7d** and **7f** are less likely to be excreted by intestinal epithelial cells because they are not P-gp substrates, and therefore it can be inferred that their passage into the systemic circulation may be more efficient.

On the other hand, the log Kp (skin permeability) values determine the skin absorption potential of the compounds, and according to the table, the calculated log Kp values for the proposed derivatives vary between −2.73 and −2.74. These skin permeability values of all compounds have a profile similar to curcumin (−2.764) and are quite low. This situation shows that the proposed compounds may not be suitable for transdermal drug application.

On the other hand, one of the most important factors affecting the absorption process is the water solubility level. Inadequate distribution of low-soluble compounds in gastrointestinal fluids may limit their oral bioavailability. In terms of solubility, the log water solubility value of curcumin is −4.01, and among the compounds in the study, there are compounds with similar solubility levels to curcumin, such as **10g** (−4.048), **7g** (−4.359), and **10a** (−4.492). This situation suggests that some of the compounds in the study have a solubility profile at least similar to curcumin in terms of formulation.

In summary, in terms of absorption properties, it is understood that the designed derivatives may have much better potential, especially in terms of intestinal permeability, compared to curcumin, and thus it can be predicted that this situation may increase their oral bioavailability.

#### 2.2.3. Distribution Properties and Tissue Penetration Potential

After absorption from the intestines, how compounds are distributed in the body is determined by pharmacokinetic parameters such as volume of distribution (VDss), plasma protein binding ratio (Fu), blood-brain barrier (BBB) permeability, and central nervous system (CNS) entry potential (log PS). At this point, it is well established that the extent to which a compound can diffuse into tissues and in which regions it can be effective is a critical factor in terms of its pharmacological efficacy [80,81,82]. The distribution properties calculated in this context are listed in Table 3.

When the table is analyzed, it is seen that the volume of distribution (log VDss) of curcumin is −0.215, which indicates that curcumin diffuses into body tissues to a limited extent and remains mostly in plasma. When the compounds in the study are examined, it can be said that they exhibit a distribution profile similar to curcumin in general, and they are compounds that largely remain in the plasma and have more limited passage to tissues.

When evaluated in terms of binding to plasma proteins (Fu), curcumin is completely bound to plasma proteins, and the free drug ratio is zero. This indicates that the circulating amount of curcumin in active form is quite limited. As seen in the table, the majority of the compounds in the study also have low free drug fractions and are largely transported bound to proteins. However, compounds such as **7a** (0.038), **7d** (0.073), **7f** (0.042), **7g** (0.044), **9a** (0.032), and **9d** (0.040) have a higher free drug fraction. These compounds may allow more active drug forms to remain in circulation and increase tissue access.

The ability to cross the BBB is a critical parameter for drugs acting on the CNS. According to the pkCSM model, compounds with log BB > 0.3 can effectively cross the BBB, while compounds with log BB < −1 are very limited in terms of entry into the brain. The log BB value of curcumin was determined as −0.562, indicating that it has the potential to enter the brain but may be limited. When the compounds in the study are evaluated, especially compound **9g** (0.231), it is the best candidate in terms of BBB permeability and stands out as a compound with potential for brain-targeted therapies. The other compounds generally have log BB values ranging from −0.2 to −0.7 and can be said to have a similar profile to curcumin.

A similar situation is observed in terms of potential CNS entry. According to the pkCSM model, compounds with log PS > −2 can effectively reach the CNS, while compounds with log PS < −3 have very limited CNS entry. The log PS value of curcumin was calculated as −2.99, which indicates that its CNS entry potential is quite low. According to the table, the CNS entry potential of all compounds proposed in the study is >−2. This situation indicates that the proposed derivatives can also be evaluated as potential candidates for neurological treatments. As a result, some compounds in the study have wider tissue distribution and higher CNS entry potential compared to curcumin, and especially compound **9g** stands out as the strongest candidate in terms of both BBB permeability and CNS entry potential. These findings indicate that some compounds in the study can overcome the limitations of curcumin and be more effective for certain pharmacological applications.

#### 2.2.4. Metabolic Stability and Cytochromes P450 (CYP) Enzyme Interactions

One of the most important stages in pharmacokinetic processes is metabolism. Metabolism ensures that compounds are converted into active or inactive forms and kept at a balanced level in the body. The metabolic stability of a compound and the enzymes with which it interacts are among the critical factors that directly determine its bioavailability, duration of action, and drug-drug interaction potential [83,84].

When the metabolic profiles of the compounds in the study were examined (Table 4), it was determined that all compounds were metabolized by the CYP3A4 enzyme. Since CYP3A4 is one of the most common cytochrome P450 enzymes involved in drug metabolism, these compounds are expected to be easily analyzed by pharmacokinetic models and to provide predictable metabolic profiles.

On the other hand, especially compounds **7d**, **7f**, **7g**, **9f**, **9g,** and **10d** may cause auto-inhibition by inhibiting the CYP3A4 enzyme. This may provide an advantage for certain compounds, allowing them to remain active in the body for a longer time and increase therapeutic efficacy. In particular, since compounds **7d**, **7f,** and **7g** have a high intestinal absorption rate and strong membrane permeability, when these properties are combined with auto-inhibition, it can be expected that they will remain in the systemic circulation longer and prolong their duration of action. Furthermore, compound **9g**, considering that it is one of the rare compounds that can cross the BBB, has the potential to remain in brain tissue for a longer time thanks to CYP3A4 auto-inhibition. This property may offer it a significant advantage in the process of developing drugs for neuroprotective or CNS diseases.

On the other hand, non-auto-inhibitor compounds, such as **9a**, **9e,** and **10a**, can control metabolic degradation and are considered candidates for long duration of action without the risk of accumulation in the body. In this respect, it can be envisaged that the balanced elimination of these compounds may contribute to maintaining stable plasma levels and reducing the risk of side effects.

Although curcumin may cause CYP3A4 auto-inhibition, it remains in systemic circulation for a short time because it is rapidly metabolized. Rapid glucuronidation, especially by UDP-glucuronosyltransferase (UGT) enzymes, is the main factor limiting its bioavailability. In contrast, some compounds in the study may have a longer duration of action because they have more controlled pharmacokinetic profiles in terms of both CYP3A4 autoinhibition and metabolic stability.

On the other hand, as seen in the table, none of the compounds proposed in the study were identified as CYP2D6 substrates. This may lead to less inter-individual variability due to CYP2D6 polymorphisms and hence more predictable pharmacokinetic profiles. In this context, it can be predicted that the inter-individual variability between slow-metabolizing and fast-metabolizing individuals, which is common for drugs metabolized by CYP2D6, will not be a significant problem for these compounds. It can be said that this is an important advantage that can increase drug safety by facilitating dose optimization for the proposed derivatives.

On the other hand, Curcumin is not an inhibitor of any of the CYP1A2, CYP2C19, and CYP2C9 enzymes, while compound **7d** is the only inhibitor of CYP1A2. When it was evaluated in terms of CYP2C19 inhibition, it was determined that all of the compounds in the study could inhibit this enzyme. At this point, it can be considered that these derivatives may have a feature that can provide therapeutic dosage advantages, especially in combination with proton pump inhibitors (e.g., omeprazole) or certain antidepressants.

When examined in terms of CYP2C9 inhibition, it was determined that all compounds except **9a**, **9e,** and **10e** can inhibit the CYP2C9 enzyme. Accordingly, it can be said that this feature may increase the therapeutic efficacy of the derivatives in combination with certain drugs and may help optimize drug regimens. Therefore, it can be expected that the potential of these compounds to regulate metabolism with certain dose adjustments may allow advantageous management of drug-drug interactions.

In summary, the compounds evaluated in this study offer significant advantages over curcumin in terms of metabolic stability, enzymatic interactions, and bioavailability. For instance, CYP3A4 auto-inhibitory curcumin derivatives can attract attention in terms of prolonged half-life and increased therapeutic efficacy, while non-auto-inhibiting compounds have the potential for safe use by exhibiting controlled pharmacokinetic profiles with stable elimination processes. Curcumin derivatives that can auto-inhibit CYP3A4 may have the potential to prolong half-life and enhance therapeutic efficacy. In contrast, those that do not exhibit CYP3A4 auto-inhibition may offer safer pharmacokinetic profiles by supporting balanced elimination and controlled metabolism. Also, the fact that they are not CYP2D6 substrates provides a more predictable clinical use by minimizing interindividual metabolic differences. In addition, thanks to their CYP2C19 and CYP2C9 inhibition properties, it is anticipated that they may offer therapeutic advantages in combination with certain drugs. In conclusion, comprehensive evaluation of the pharmacokinetic profiles of these derivatives in the study may contribute to the creation of more effective and safer treatment strategies in drug development processes.

#### 2.2.5. Excretion Profiles and Clearance Characteristics

The removal of metabolized compounds from the body is one of the main factors determining the duration of action of drugs. The excretion process is largely related to total clearance (log mL/min/kg) and renal transport systems (OCT2 substrate) [85,86,87]. Table 5 shows the calculation results of the compounds based on these relevant parameters. According to the table, the total clearance value of curcumin is −0.002 log mL/min/kg, and this indicates that the elimination rate is quite low. This suggests that curcumin may remain in systemic circulation for a long time, and its bioavailability may be prolonged. Among the compounds in the study, **10b** (−0.227) and **5g** (−0.219), which have the lowest clearance values, similarly stand out as candidates that may show a long duration of action.

On the other hand, most of the compounds in the study have higher clearance values compared to curcumin. These results indicate that drug elimination rates of the derivatives may be higher and the risks of accumulation in the body may be reduced. Especially compounds such as **9a** (0.194) and **9e** (0.183) stand out with their high clearance values. Overall, it can be inferred that the rapid elimination profile of the majority of the designed derivatives indicates that they have the potential to minimize the risk of unwanted drug accumulation in long-term use.

In addition, curcumin and none of the compounds in the study were identified as OCT2 substrates. In this context, it can be said that the compounds are primarily eliminated via hepatic metabolism and can be eliminated from the body via bile. This situation suggests that the ability to provide metabolic excretion without renal elimination dependency may offer the advantage of safe use of these compounds, especially in patients with renal insufficiency. When excretion characteristics are evaluated in general, some compounds in the study tend to remain in the body longer with low clearance values, which may provide advantages for long-term pharmacological activity, while some compounds show faster elimination with high clearance levels, reducing the risk of drug accumulation. Accordingly, it can be said that this dual advantage may allow dose adjustments according to different therapeutic needs and flexibility in drug development processes.

#### 2.2.6. Toxicological Evaluation: Safety Profile and Risk Assessment

As it is well established that the toxicity profiles of metabolized compounds are one of the most critical factors to determine their safe use range and the potential side effect risks [88,89,90]. In this context, in the presented study, genotoxicity (AMES test), maximum tolerated dose (MTD), cardiotoxicity (hERG inhibition), acute and chronic toxicity, hepatotoxicity, and environmental toxicity parameters (Minnow and T. Pyriformis toxicity) were addressed in the toxicological evaluation, and the results obtained are presented in Table 6.

Accordingly, it is seen in the table that curcumin gave negative results in the AMES test, did not show hERG channel inhibition, and was not hepatotoxic. When it is examined in the table, compounds **7a**, **7d**, **7f**, **7g**, **9a**, **9e**, and **10g** gave negative results in the AMES test, and this indicates that the risk of genotoxicity is low. This supports their potential as reliable candidates for long-term use. Furthermore, no hERG I channel inhibition was detected in any of the compounds in the study. This characteristic corresponds to a low risk of cardiotoxicity, and thus it increases their potential for the compounds to be safe in clinical use. In terms of hepatotoxicity, compounds **7a**, **9f**, **10f**, and **10g** did not exhibit hepatotoxic effects. This feature stands out as an important advantage in pharmacological applications, where liver toxicity is a major clinical concern.

On the other hand, it can be observed that the proposed derivatives in the study offer various advantages compared to curcumin. Accordingly, when it is evaluated in terms of MTD, some compounds have higher MTD values compared to curcumin. Particularly, compounds **7d** (0.366), **7a** (0.258), and **9a** (0.270) offer a wider safety frame with higher MTD values. This indicates that these compounds can be used at higher doses, and the risk of toxicity may be lower.

Regarding oral acute toxicity, it is seen that derivatives have higher LD50 values than curcumin. Especially, compounds **9a** (3.107), **9e** (3.004), and **10g** (3.834) exhibit the most favorable toxicity profiles, with higher LD50 values than curcumin. At this point it can be said that these derivatives may provide advantages in the drug development process due to their higher safety margins.

Similarly, in terms of chronic toxicity by oral route (LOAEL), all derivatives have higher LOAEL values compared to curcumin. Notably, compounds **7a** (1.404), **7d** (2.150), and **9a** (1.502) show lower toxicity risk for long-term use with higher LOAEL values. This suggests that these compounds may be more advantageous in diseases requiring chronic treatment.

According to environmental toxicity results, the T. Pyriformis toxicity values (−0.29 to −0.305 log µg/L) of the compounds in the study are at similar levels to curcumin, and this situation demonstrates that they are low-risk compounds in terms of the environment. Regarding minnow toxicity, the compounds exhibit a better profile compared to curcumin, and especially compounds such as **10e** (−1.005), **10f** (−1.124), and **10g** (−2.312) show significantly lower toxicity. This provides these compounds with a notable advantage in terms of environmental sustainability.

### 2.3. Molecular Docking

A molecular docking study was conducted to ascertain the binding affinities of 19 novel curcumin derivatives, which possess a more reliable ADMET profile and demonstrate the potential to serve as efficacious drug candidates, to the PLpro enzyme structures of SARS-CoV, MERS-CoV, and SARS-CoV-2, which are responsible for severe respiratory diseases and pandemics among the seven coronavirus species detected in humans. In order to facilitate a comprehensive comparison of the binding affinities obtained, additional docking studies were performed on the PLpro enzyme target structures of these three distinct CoVs for natural curcuminoids (curcumin, bisdemethoxycurcumin, and demethoxycurcumin), various reference drugs used in the treatment of COVID-19, such as favipiravir, hydroxychloroquine, lopinavir, and warfarin, and the PLpro-specific inhibitor VIR250. Accordingly, the affinity values of each compound at the optimal binding position to the target enzymes were calculated and listed in Table 7.

Analyzing the docking values obtained for the PLpro target enzyme structure (6WUU) of the SARS-CoV-2 virus, the 19 new curcumin derivatives proposed in the study have a higher binding affinity than natural curcuminoids, all reference drugs, and the enzyme inhibitor VIR250. This situation confirms the potential inhibitory effect of the proposed compounds on PLpro of SARS-CoV-2 and their strong binding capacity. Furthermore, when the results obtained for 19 new curcumin derivatives proposed in this study for the 6WUU structure are compared with the results obtained for 22 different curcumin derivatives proposed in our previous study, it is observed that 12 of the 19 compounds (excluding **5g** (−9.5 kcal/mol), **6g** (−9.4 kcal/mol), **7a** (−9.3 kcal/mol), **7f** (−9.5 kcal/mol), **9e** (−9.3 kcal/mol), and **9f** (−9.1 kcal/mol)) were found to have at least as high a binding affinity compared to the curcumin derivatives proposed in the previously reported study, in which top-scoring compounds had an affinity of −9.6 kcal/mol. Moreover, the curcumin derivatives with the highest docking scores for the SARS-CoV-2 PLpro target enzyme in this study were **10d** (−10.0 kcal/mol) and **10g** (−10.1 kcal/mol). As a result, it is very clear that the binding affinity results obtained for the PLpro enzyme of the SARS-CoV-2 virus associated with the COVID-19 outbreak have been improved with the proposed new curcumin derivatives.

Here, comparison of docking results with PLpro enzymes of other CoVs is also critical for understanding binding trends between species. In this context, when the binding affinities obtained for SARS-CoV-2 PLpro (6WUU) are compared with the affinities calculated for SARS-CoV PLpro (2FE8) and MERS-CoV PLpro (4RNA), it is seen that the binding scores for MERS-CoV PLpro are slightly lower than the other ones. However, it is noteworthy that the affinity values obtained for SARS-CoV PLpro were significantly higher compared to SARS-CoV-2 PLpro.

Consequently, it can be said that these results suggest that the proposed 19 novel curcumin derivatives may have a strong inhibitory effect on the PLpro enzyme structures of three different CoVs detected in humans. Accordingly, it can be inferred that the high-affinity binding of curcumin derivatives to the PLpro enzymes of pandemic-causing viruses such as SARS-CoV, MERS-CoV, and SARS-CoV-2 strengthens the possibility that these compounds may also be effective against new coronavirus strains that may emerge in the future.

**Figure 2:**
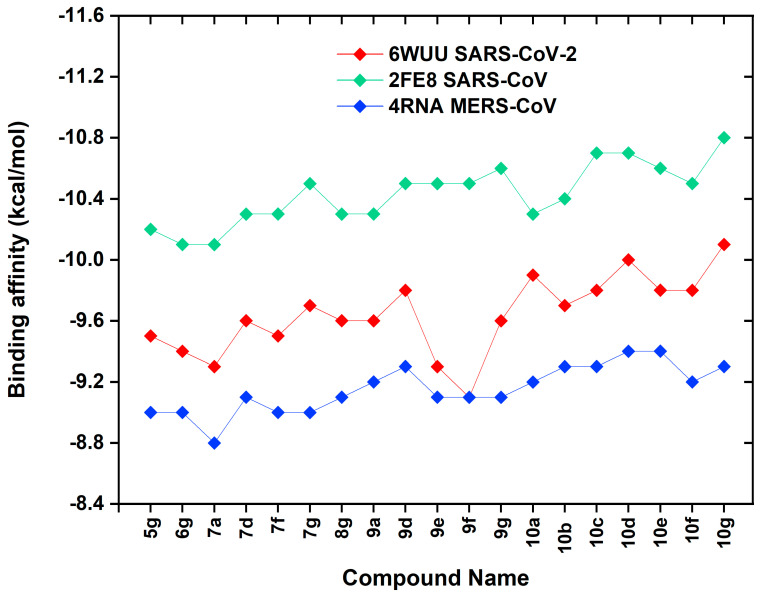
Comparison of Binding Affinities of Curcumin Derivatives Against Different Coronavirus PLpro Enzymes.

**Figure 3:**
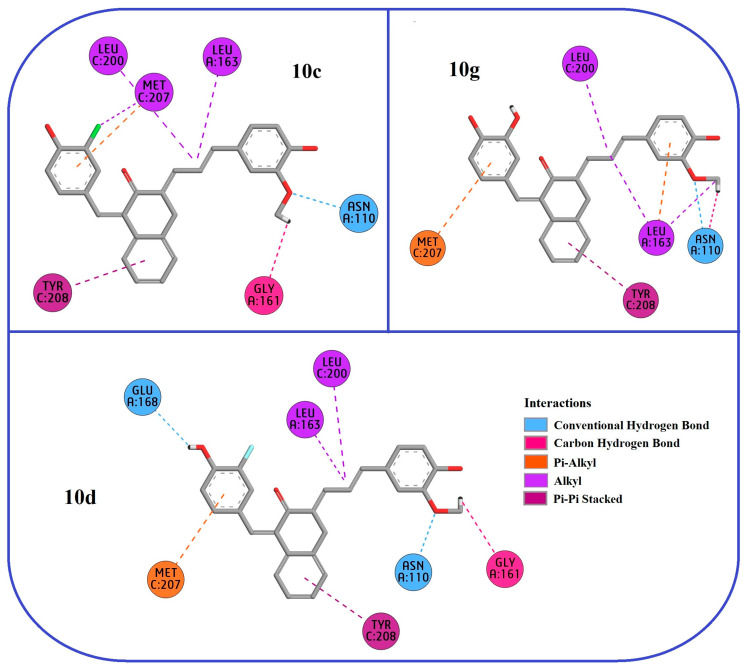
2D Interaction Diagrams of the Top-Scoring Compounds (**10c**, **10d**, and **10g**) with SARS-CoV PLpro Enzyme.

**Figure 4:**
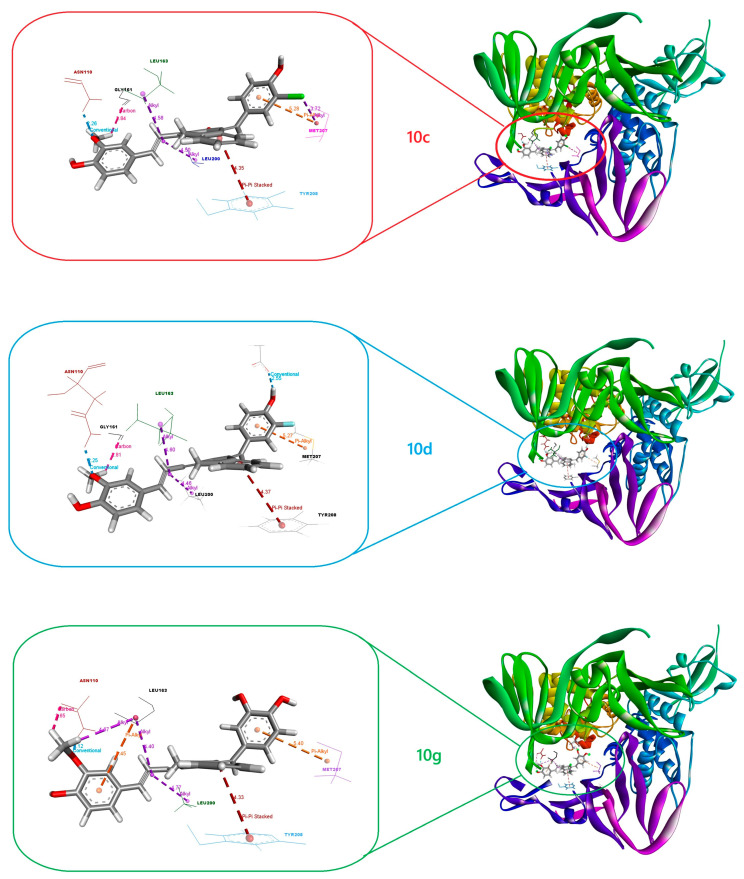
3D Interactions of the Top-Scoring Compounds (**10c**, **10d**, and **10g**) with SARS-CoV PLpro Enzyme.

In order to better visually compare the binding affinity trends of curcumin derivatives on PLpro enzymes, the obtained docking scores are presented graphically in Figure 2. When this graph is analyzed, it is seen that the binding trends of curcumin derivatives to the PLpro enzymes of CoVs generally follow a sequence as SARS-CoV (2FE8) > SARS-CoV-2 (6WUU) > MERS-CoV (4RNA). In particular, the three curcumin derivatives that bind most strongly with SARS-CoV PLpro (2FE8) were **10g** (−10.8 kcal/mol), **10c** (−10.7 kcal/mol), and **10d** (−10.7 kcal/mol), respectively. In this context, 2D interaction diagrams were constructed to elaborate the binding mechanisms and molecular interactions of the hit compounds **10c**, **10d,** and **10g**, which have the highest affinity with the SARS-CoV PLpro structure, and are presented in Figure 3. Also, 3D interaction details were depicted in Figure 4.

When the interaction diagram was examined, it was understood that compounds **10c** (−10.7 kcal/mol), **10d** (−10.7 kcal/mol), and **10g** (−10.8 kcal/mol), which showed the highest binding affinity with the SARS-CoV PLpro enzyme, exhibited strong inhibitory properties thanks to a hydrogen bond, hydrophobic interactions, and aromatic ring interactions with critical amino acids in the active site. At this point, common amino acid residues with which these three compounds interact can be considered as key residues regulating the inhibitor binding of the enzyme and can be used as strategic targets in new inhibitor designs.

In this regard, examining the figure in detail, especially ASN110, shows direct interaction with the active site by forming hydrogen bonds with all three compounds. Here, ASN110, which forms a conventional hydrogen bond (coHB) with compounds **10c** and **10d**, stands out as an important residue that can play a key role in inhibitor binding by forming both a conventional hydrogen bond and a carbon hydrogen bond (caHB) with compound **10g**. In addition, LEU163 and LEU200 exhibit hydrophobic alkyl interactions with all three compounds, which can be said to provide more stable binding of inhibitors in the active site. In particular, while compounds **10c** and **10d** form one alkyl interaction with LEU163, compound **10g** exhibits a stronger hydrophobic interaction by forming two alkyl and one Pi-alkyl interaction. Accordingly, it can be said that this situation indicates that LEU163 can be a significant hydrophobic attraction center for the PLpro binding site.

Furthermore, MET207 stands out as another critical amino acid residue that enhances binding stability at the active site by exhibiting Pi-alkyl and alkyl interactions with all three compounds. Moreover, a common Pi-Pi stacked interaction with all three compounds was observed with TYR208. This interaction can contribute to the stabilization of the inhibitors at the enzyme active site by promoting strong binding between aromatic rings, and hence, it can be considered that TYR208 may be another key residue that plays a critical role in inhibitor binding. On the other hand, GLY161 may function as one of the additional stabilization points between the ligand and the enzyme surface by forming caHB with compounds **10c** and **10d**.

**Figure 5:**
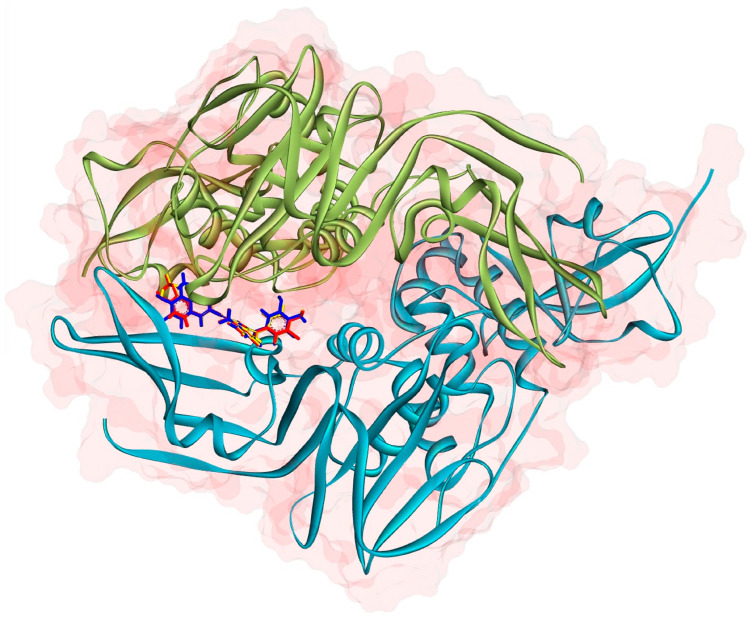
3D Binding Orientation of the Hit Compounds (**10c**, **10d**, and **10g**) in the Active Site of Dimeric SARS-CoV PLpro Enzyme.

In light of these findings, the presence of these common interactions suggests that compounds **10c**, **10d,** and **10g** bind to the same active site on the SARS-CoV PLpro enzyme and may have a common inhibitory mechanism. Furthermore, the positioning of these compounds at approximately the same binding site suggests that the enzyme inhibition mechanism is based on a common model. For a better visual depiction of this situation, the 3D binding positions of **10c**, **10d,** and **10g** on the dimeric SARS-CoV PLpro enzyme are presented in Figure 5. As can be seen from the figure, the compounds bind to the same active site as expected and have almost the same binding orientation. Although the current findings offer a detailed computational perspective, experimental validation through biochemical and cellular assays remains essential to confirm the predicted inhibitory activities and pharmacokinetic behaviors of the proposed compounds.

### 2.4. MD Simulations and MM-PBSA Analysis

Molecular dynamics (MD) simulations are one of the most suitable theoretical tools to evaluate the interaction dynamics and binding stability of the hit compounds identified as a result of docking studies with the SARS-CoV PLpro enzyme in the environment closest to physiological conditions. Although docking analyses play a critical role in determining the binding affinities of ligands to the protein, they cannot fully reflect the dynamic response of the protein after ligand binding. Therefore, MD simulations are needed to be performed to examine the effect of hit compounds on the conformational flexibility of PLpro in more detail and to evaluate the stabilization of the protein after binding. Here, in addition to the MD simulations between the hit compounds and SARS-CoV PLpro, MD simulations were also conducted for the wild-type (WT) form of PLpro to better understand how ligand binding alters the native mobility of the protein.

**Figure 6:**
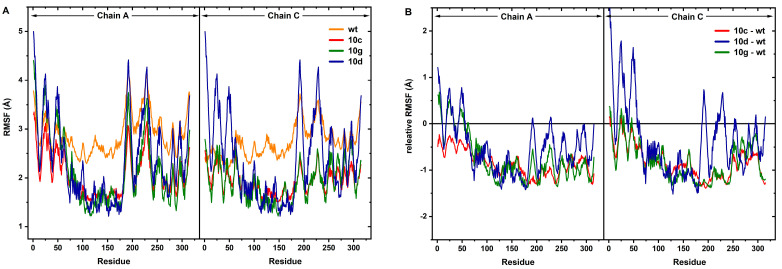
(**A**) RMSF and (**B**) Relative RMSF Analyses from MD Simulations for SARS-CoV PLpro–Ligand Complexes.

In this regard, Root Mean Square Fluctuation (RMSF) was first computed using the productive phase data obtained from MD simulations. As is known, this analysis is used as a critical parameter to locally assess the effect of ligand binding on the dynamic structure and to examine the flexibility changes in specific regions of the protein in detail. Here, considering the dimeric structure of PLpro, RMSF calculations were performed separately for both monomers (Chain A and Chain C) in order to examine the potential effects of ligands on binding sites in detail (Figure 6A). Furthermore, relative RMSF analysis was performed to depict more clearly how ligand-bound systems change the flexibility of the protein. In this analysis, the relative effect of ligand binding on the overall mobility of the protein was evaluated by subtracting the RMSF values calculated for WT from the RMSF values per residue obtained for the ligand systems (Figure 6B). This comparative analysis allows a clearer evaluation of the effects of the identified hit compounds on the active site and other functional regions of the protein, while it can also contribute to a better understanding of the potential inhibitory mechanisms of the ligands.

When the RMSF data obtained were analyzed, it was observed that the stability of the protein generally increased in all hit compound-bound systems compared to WT. Especially in the 75–250 residue range, it is observed that the RMSF values of the hit compound-bound systems decreased significantly compared to WT. This situation attributes that the binding of hit compounds suppresses the overall flexibility of the protein and creates a more rigid structure. In this context, significant RMSF decreasing in RMSF values was observed in the loop regions around the active site, especially in areas containing MET207, TYR208, LEU200, ASN110, and GLU168 in ligand-bound systems. The decrease in mobility in these regions indicates that the ligand may increase inhibitory activity by suppressing enzyme activity.

In addition, in the WT system, residue regions 155–170, 215–240, and 265–280, known as the dimerization region of PLpro, showed slightly lower RMSF values compared to the other residue regions. In this context, it can be said that inter-monomer interactions are inherently stable. In the ligand-bound systems, it was observed that the RMSF decrease in these regions continues, and thus the dimerization interface does not undergo a major structural change after ligand binding.

When the stabilization effects of the hit compounds compared to each other were evaluated, it was determined that **10g**, which has the lowest binding energy in docking analyses, has the lowest RMSF value trend and stands out as the compound that stabilizes the protein the most. On the other hand, it can be noted that although **10d** showed a slightly higher RMSF value trend compared to **10g**, it provides a significant stabilization compared to WT and decreases the overall mobility of the protein. In addition, **10c** has the highest RMSF value trend amongst the hit compounds and appears to be the compound that least stabilizes the enzyme compared to the other two compounds. This situation suggests that the inhibitory effect of **10c** on PLpro may be lower compared to **10g** and **10d**.

In summary, when the results of docking and MD simulations were evaluated together, it can be stated that **10g** was the strongest inhibitor candidate because it had the lowest binding energy and showed the lowest RMSF values in MD simulations. Also, compound **10d** is an intermediate candidate in terms of stabilization, while compound **10c** appears to be the weaker candidate in terms of both binding affinity and dynamic stabilization. Furthermore, no significant increase in post-binding mobility was observed at the dimerization site, and hence, these findings show that hit compounds do not disrupt monomer-monomer interactions of the PLpro and preserve its dimer structure. Accordingly, it can be inferred that these analyses show that not only the binding strength but also the dynamic response of the protein after binding should be considered in PLpro inhibitor design.

The RMSF analyses obtained after MD simulations allowed a detailed evaluation of the stabilization effects of the hit compounds on PLpro. However, in order to evaluate the inhibitory activity of a ligand more comprehensively, it is necessary to calculate the free energy changes after binding. In this context, the binding free energies of ligands were calculated using the MM/PBSA method, and the thermodynamic profile of ligand-protein interactions was analyzed. In this regard, it is well established that the MM/PBSA method is a widely used computational approach to compare the binding strength and potential inhibitory activities of ligands by determining the binding free energy of ligand-protein complexes. The method incorporates various components, including van der Waals (Δ*G*^vW^), electrostatic (Δ*G*^elec^), polar (Δ*G*^ps^), and non-polar solvation (Δ*G*^nps^) energies, which are taken into account during the calculation of the binding free energy (Δ*G*^bind^).

In this respect, the binding free energy and components of hit-scoring curcumin derivatives were listed in Table 8. When the calculated MM/PBSA binding free energies were scrutinized, it was observed that all compounds bind strongly to the protein, and significant differences were detected between the binding energies. Especially compound **10g** has the lowest binding free energy (Δ*G*^*bind*^ = −280.72 kJ/mol) and stands out in terms of binding stability. This finding is also consistent with the highest binding affinity observed in docking analyses. In particular, it is, herein, clear that **10g** receives the largest contribution from the interactions Δ*G*^vw^ = −342.89 kJ/mol and Δ*G*^elec^ = −112.75 kJ/mol. In addition, the energy of Δ*G*^ps^ (+165.98 kJ/mol) was observed to be the highest compared to the other compounds, and this indicates that the solubility of the ligand in a water medium is increased. However, this is a factor that reduces the binding stability. On the other hand, compound **10d** showed a high binding stability with a binding free energy of Δ*G*^*bind*^ = −268.85 kJ/mol. Here, it is seen that the electrostatic interactions (−105.23 kJ/mol) and van der Waals interactions (−328.67 kJ/mol) of **10d** remain at a lower level compared to compound **10g** ones. In contrast, although compound **10c** showed an effective binding with a binding free energy of Δ*G*^*bind*^ = −259.43 kJ/mol, it exhibited a more moderate stabilization profile compared to the other two hit compounds. At this point, it was seen that its values of Δ*G*^vw^ = −315.12 kJ/mol and Δ*G*^elec^ = −98.65 kJ/mol remained at a lower level compared to the other two hit compounds.

It is also clear that the dominant interaction types contributing to the binding free energy for the three hit compounds are van der walls interactions.

These findings show that MM/PBSA analyses are in agreement with docking and MD simulations and confirm that hydrophobic interactions and electrostatic bonds play a critical role in inhibitor design. In particular, the best stabilization of **10g** supports it as the most potent inhibitor candidate, while **10d** and **10c** can also be evaluated as compounds with remarkable inhibitory potential.

Thanks to MM/PBSA calculations, amino acid residues that contribute positively to ligand binding or maintain their mobility after binding can be determined. In this context, hot residues can be defined as amino acids that become stable following ligand binding and play a key role in the binding process. On the other hand, unhot residues can be considered as residues that maintain their mobility during ligand binding and provide a more limited contribution to binding stabilization. Here, a classification can be made on per-residue energy contributions, and here, residues with a contribution to the binding energy lower than −2.00 kJ/mol (i.e., more negative) were considered hot, while those higher than +2.00 kJ/mol were considered unhot. These thresholds were chosen similarly as in the literature to assess whether the energetic contributions in the binding site are significant.

Furthermore, in order for an amino acid to be classified as “hot” or “unhot” in the study, it is required that the relevant residue meet the relevant criteria in at least two of the three different hit compounds that bind strongly. The approach adopted here aims to identify residues that play a truly critical role in binding while avoiding random variations. Residues determined as hot or unhot, along with their energetic values, are listed in Table 9.

Accordingly, Asn110, Gly161, Glu162, Leu163, Asp164, Glu167, Thr198, Leu200, Val203, Met207, Tyr208, Ser245, Tyr268, Tyr269, and Tyr273 were observed as hot residues. Here it can be said that these residues, which became stable after binding, directly contributed to the inhibitor stability and supported the effective binding of the ligand by creating rigidity in the enzyme structure. Especially Asn110, Gly161, Glu162, Leu163, and Asp164, which are located close to the binding site of PLpro, are among the best critical residues that become stable by establishing hydrogen bond interactions with the ligands. Notably, it is inferred from the obtained values in the table that Glu162 and Leu163, herein, provided the highest stability after binding, increased the rigidity of the protein, and strengthened the inhibitor binding. Another residue whose rigidity increased after binding was Glu167. Here, it can be said that this residue, which can support ligand binding through electrostatic interactions, is an important component that directly affects inhibitor binding. Also, Thr198 and Leu200 are among the residues with the most decreased mobility after binding. The fact that the energy levels are below −2 kJ/mol indicates that these residues gain conformational rigidity after binding and become more stable in the enzyme structure. Additionally, Val203, Met207, and Tyr208 were determined as important residues that support inhibitor binding by providing post-binding stabilization through hydrophobic interactions and π-π stacking. Here it can be expressed that while the large van der Waals surface of Met207 can support ligand binding, Tyr208 can contribute to ligand stability with its aromatic surface. On the other hand, for Ser245 and Tyr268 in the binding region, it can be noted that they became stable through hydrogen bonding after ligand binding. Moreover, it can be stated that Tyr269 and Tyr273 were included among the regions that gain rigidity after binding despite being located close to the dimerization region. Moreover, the low RMSF (root mean square fluctuation) values observed at these hot residues throughout the MD simulations indicate that the mobility after binding is significantly reduced, and inhibitor binding stabilizes the PLpro enzyme.

When the ligands were compared to each other according to the binding stability, it was seen that compound **10g** provided the strongest stabilization effect by interacting with the hot residue the most. Especially Asn110, Gly161, Glu162, Glu162, Leu163, Met207, and Asp164 were stabilized after binding, supporting the tight binding of the ligand to the enzyme. Additionally, it may be interpreted that compound **10d** showed the effect of inhibitor binding on enzyme dynamics by increasing post-binding rigidity, especially at sites such as Met207, Tyr208, and Ser245. On the other hand, although compound **10c** provided relatively lower stabilization compared to the other two compounds, an increase in post-binding rigidity was observed at sites such as Asn110, Gly161, Tyr208, and Tyr273.

On the other hand, Arg82, Lys108, Lys157, Glu167, Arg183, Glu204, Lys232, Pro248, Tyr264, and Asp302 appeared as unhot residues as seen in the table.

In summary, these findings are an important guide for PLpro inhibitor design and provide valuable information on how to optimize the binding regions. Taken together, the present findings highlight the significant potential of the proposed curcumin derivatives as broad-spectrum PLpro inhibitors. Compared to curcumin and existing antiviral agents, the selected derivatives exhibited superior pharmacokinetic properties, higher binding affinities, and improved structural stability. Importantly, the consistent inhibitory trends across PLpro enzymes of SARS-CoV, MERS-CoV, and SARS-CoV-2 suggest a conserved binding mechanism, strengthening their potential against future emerging coronavirus strains. These results provide a strong foundation for future experimental validation and the possible development of orally available antiviral therapies. PLpro enzymes are known not only for their role in polyprotein cleavage during viral replication but also for their ability to suppress host innate immunity by deISGylating ISG15 and cleaving ubiquitin-like modifiers. Therefore, the inhibition of PLpro may simultaneously block viral propagation and restore interferon-mediated immune responses. The structural conservation of the PLpro catalytic domain across SARS-CoV, MERS-CoV, and SARS-CoV-2 supports the potential of these curcumin derivatives as broad-spectrum antivirals, capable of remaining effective even in the face of emerging viral variants. By occupying the catalytic triad and forming strong hydrogen bonds and hydrophobic interactions with key residues (e.g., Tyr269, Gln270, Asp165, and Leu163 in SARS-CoV PLpro), the designed curcumin analogs likely act as competitive inhibitors that prevent substrate cleavage and enzymatic activation. Furthermore, the identification of key hotspot residues can guide the rational design of next-generation PLpro inhibitors with enhanced efficacy and selectivity.

Recent computational studies published in 2024 and 2025 have similarly explored diverse synthetic scaffolds and natural products as PLpro inhibitors, employing molecular docking, MD simulations, and AI-driven modeling approaches [91,92,93,94,95]. These findings are in alignment with our results and reinforce the relevance of targeting PLpro for broad-spectrum antiviral drug design.

The promising in silico performance of the proposed curcumin derivatives in targeting PLpro is particularly noteworthy given the increasing attention to alternative protease targets beyond 3CLpro. While several compounds such as ensitrelvir and simnotrelvir have advanced as potent 3CLpro inhibitors [36,41], comparatively fewer candidates have been developed to specifically inhibit PLpro. Additionally, nucleoside analogs like molnupiravir, azvudine, and remdesivir continue to demonstrate efficacy by inhibiting RdRp, yet they often suffer from cytotoxicity or limited bioavailability [37,38,39]. In contrast, various natural products—such as cordycepin, riboprine, and phytochemicals derived from ginger and pineapple—have been computationally predicted to inhibit multiple SARS-CoV-2 enzymes; however, only a few have progressed to clinical validation [39,42,50]. Our results suggest that the designed curcumin derivatives not only display strong binding affinity to PLpro enzymes of SARS-CoV, MERS-CoV, and SARS-CoV-2, but also exhibit favorable ADMET and drug-likeness profiles in line with or exceeding those of previously studied gallates, oxadiazoles, and isoquinolines [34,35,37]. Furthermore, the proposed derivatives provide a potentially safer and more synthetically accessible alternative to existing synthetic antivirals. Thus, this work contributes to filling a critical gap in anti-SARS-CoV-2 research by proposing novel compounds with multi-variant PLpro inhibition potential and favorable pharmacokinetic properties.

## 3. Materials and Methods

### 3.1. ADMET Predictions

Firstly, the 2-D structures of the compounds were drawn using the Avogadro program, and the structures were optimized using the MMFF94s force field in this program [96]. Then, ADMET analyses were performed to evaluate the pharmacokinetic compatibility and potential toxicity of 42 new curcumin derivatives. As a result of these analyses, further analyses were continued with 19 compounds with higher potential by filtering according to Ro5 rules in the first stage [58]. Comprehensive ADMET predictions in the study were calculated using SwissADME [97], pkCSM [98], ADMETLAB2.0 [99], and PredHERG5.0 [100] platforms.

### 3.2. Molecular Docking Calculations

The three-dimensional structures of PLpros were retrieved from the Protein Data Bank (PDB) database for the major coronavirus species that have caused severe pandemics in humans, including SARS-CoV (PDB ID: 2FE8 [16]), MERS-CoV (PDB ID: 4RNA [101]), and SARS-CoV-2 (PDB ID: 6WUU [13]).

Molecular docking was carried out using AutoDock Vina 1.1.2 software [102]. The binding sites were optimized based on previous experimental and computational studies, and in this context, the grid box dimensions were adjusted to cover the active site for each protein. Accordingly, the grid boxes for SARS-CoV, MERS-CoV, and SARS-CoV-2 were determined as 40 × 40 × 40 Å, 45 × 45 × 45 × 45 Å, and 50 × 50 × 50 × 50 Å, respectively. In addition, the exhaustiveness parameter was set to 32 to ensure that the binding site was scanned over a wider range in all docking operations.

Protein structures were processed using AutoDockTools 1.5.6 [103], and here, solvent molecules and water molecules bound to the crystal structure were removed. In addition, co-factors and metal ions bound to the crystal structure were removed from the system as they were not necessary for docking analysis, polar hydrazenes were added, and Gasteiger charges were calculated. Then, ligand structures were optimized using Open Babel 3.1.1 [104] and converted to the appropriate PDBQT format, and flexible binding sites were defined by determining the rotating bonds. Thus, proper preparation of proteins and ligands was ensured prior to docking simulations.

**Figure 7:**
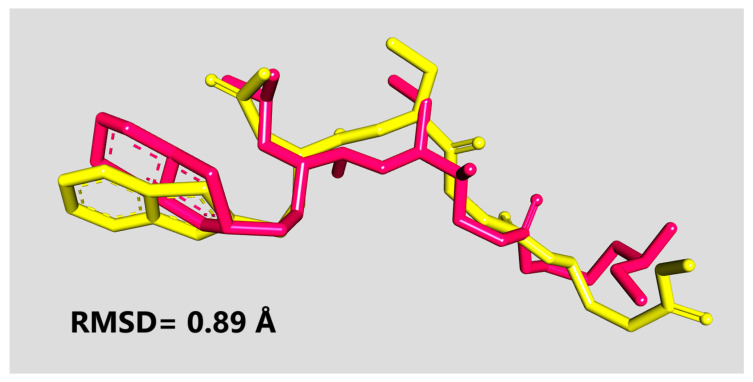
Redocking validation of the native ligand into the binding site of (PDB ID: 6WUU). The co-crystallized pose (yellow) and the re-docked pose (magenta) are superimposed to assess binding pose reproducibility.

As a result of docking calculations, 9 different binding conformations were generated for each enzyme-ligand complex, and the complexes with the lowest binding energy of them were selected and subjected to detailed analyses. In addition, in order to verify the validity of docking, re-docking was performed for each protein using reference inhibitors bound to the existing crystal structure, and the binding positions were compared with the crystal data. In order to determine the validation of the docking algorithm, the original crystal inhibitor and the re-docked positions were superimposed, and the Root Mean Square Deviation (RMSD) values were calculated. At this point, as it is known, docking results with RMSD values below 2.0 Å can be considered valid [20,105]. In our redocking validation, the RMSD between the co-crystallized ligand and its re-docked pose was found to be 0.89 Å, confirming the accuracy of our docking protocol for the 6WUU structure (Figure 7). Moreover, the obtained docking results were analyzed using Discovery Studio Visualizer to visualize protein-ligand interactions.

### 3.3. MD Simulation Protocol

MD simulations were performed between the hit compounds identified by docking calculations and the SARS-CoV papain-like protease (PLpro, PDB ID: 2FE8), in which they are more active. Thus, it was aimed to evaluate the binding stability of the hit compounds, conformational changes in the enzyme, and dynamic interactions of the complexes.

Simulations were performed using GROMACS 2020.1 software [106]. Firstly, the free (WT) form of SARS-CoV PLpro and the holo complexes of SARS-CoV PLpro (hit compound bound states) obtained by docking were prepared for simulations. At this stage, while protein structures were modeled with the CHARMM36m force field [107], the topology and parameters of the ligands were derived using CGenFF (CHARMM General Force Field) [108,109]. Then, these systems were placed in a cubic simulation box using the TIP3P water model, and here solvation was performed by leaving at least a 12 Å water layer from the protein surface. The simulation box was defined with dimensions of 10 nm × 10 nm × 10 nm × 10 nm so as not to limit the natural movements of the system. Furthermore, periodic boundary conditions (PBC) were applied in all three dimensions to allow continuous system behavior and eliminate edge effects. In addition, the systems were modeled at physiological temperature (310 K), atmospheric pressure (1 atm), and 0.15 M ion concentration conditions. In this context, in accordance with the PME (Particle Mesh Ewald) method [110], Na⁺ and Cl^−^ ions were added at a concentration of 0.15 M NaCl in order to neutralize the total charge of the system and to provide physiological environmental conditions.

Afterwards, the neutralized system was subjected to two-stage energy minimization. In the first stage, a more stable structure was created by minimizing the initial high-energy regions of the system with the steepest descent algorithm. In the second stage, a more precise energy minimization was performed using the conjugate gradient algorithm, and the system became more stable in local energy troughs.

After energy minimization, the system was subjected to two-stage equilibration simulations for a total of 4 ns. First, the temperature was raised to 310 K under the NVT ensemble, and the system stabilized for 2 ns. Then, the pressure was fixed to 1 atm and the temperature to 310 K under the NPT ensemble, and the system was subjected to pressure and temperature equilibration for 2 ns.

After equilibration, the system was carried out to production simulations under NPT conditions for 100 ns. During the simulation, long-range electrostatic interactions were calculated using the PME (Particle Mesh Ewald) [110] method, and hydrogen-containing bonds were stabilized with the SHAKE algorithm [111]. Also, a time step of 2 fs was used in the simulations, and during the simulation, coordinate, velocity, and energy data were recorded every 10 ps for detailed analyses.

In all equilibration and production simulations, temperature control was carried out with the v-rescale thermostat [112], while pressure stabilization was achieved using the Parrinello-Rahman barostat [113]. At this point, the coupling constants were set as τ^T^ = 0.1 ps for temperature and τ^P^ = 2.0 ps for pressure. The RMSD trajectories of the protein-ligand complexes were monitored throughout the simulation. The plots (Figure S1, Supplementary Materials) confirm that the systems reached convergence after approximately 10–15 ns, indicating good structural stability. However, one limitation of the present work is that the MD simulations were conducted for 100 ns per complex; future studies may consider longer simulation times (e.g., 200 ns or more) to capture slower conformational changes and validate long-term complex stability.

### 3.4. MM/PBSA Free Energy Calculations

Following detailing the dynamic effects of hit compounds on the SARS-CoV PLpro active site using MD simulation data, binding free energies were calculated using the MM/PBSA method to evaluate the binding stability of hit compounds. The g_mmpbsa module developed by Rashmi Kumari and colleagues [114] was used in the calculations. Accordingly, the binding free energy for the protein-ligand complex is calculated using the MM/PBSA approach as follows.

(1) $${\Delta E}_{MM/PBSA}=E_{complex}-(E_{protein}+E_{ligand})$$

Here, E_complex_ represents the total MM/PBSA binding energy of the complex, while E_protein_ and E_ligand_ denote the total free energy of the isolated protein and ligand, respectively. Each energy term E_i_ can be expressed as follows:

(2) $$E_{i}=E_{MM}+E_{solv}$$

Here, E_MM_ is the molecular mechanical energy and is the sum of the contributions of the electrostatic and van der Waals energy terms. Esol are the energy contributions due to the solvent and are the sum of the polar and apolar contributions. In this study, binding energies were calculated by neglecting the entropic contributions according to the MM/PBSA method.

In the study, MM/PBSA calculations were analyzed over a total of 400 frames taken at 100 ps intervals in the last 40 ns time obtained from MD simulations. In the energy calculations, the internal dielectric constant was assigned as 2 and the external dielectric constant as 80 to reflect the dielectric differences between the protein internal environment and the solvent external environment. A grid size of 0.5 Å was used for the Poisson-Boltzmann equations, and a probe radius of 1.4 Å was used for the apolar solvation contribution.

## 4. Conclusions

This study presents a rational and systematic approach to the development of novel curcumin derivatives with enhanced antiviral potential, specifically targeting the highly conserved PLpro enzyme found in major human-pathogenic CoVs: SARS-CoV, MERS-CoV, and SARS-CoV-2. By applying a robust in silico pipeline encompassing rational molecular design, ADMET-based filtering, molecular docking, MD simulations, and MM/PBSA free energy calculations, 42 novel curcumin derivatives were generated, of which 19 met key drug-likeness and pharmacokinetic criteria.

Notably, docking results revealed that these derivatives exhibit significantly improved binding affinity compared to both native curcumin and reference antiviral drugs (e.g., remdesivir, lopinavir, and hydroxychloroquine). Among the three PLpro targets, the compounds consistently showed the strongest binding affinities and most stable interactions with the SARS-CoV PLpro (PDB ID: 2FE8) enzyme. Particularly, compounds 10g, 10d, and 10c displayed the highest binding energies, coupled with key stabilizing interactions such as hydrogen bonding with ASN110 and π–π stacking with TYR208, both of which are known to be critical residues in PLpro’s active site.

Moreover, molecular dynamics simulations demonstrated that these top-scoring compounds significantly reduced local residue fluctuations in the active site and surrounding loop regions, contributing to increased structural rigidity and suggesting effective inhibition of enzymatic activity. MM/PBSA analyses further supported these findings, with compound 10g showing the most favorable free binding energy profile, driven largely by van der Waals and electrostatic interactions.

In addition to their strong inhibitory profiles, the selected derivatives showed superior pharmacokinetic properties, including high intestinal absorption, favorable CNS penetration potential, metabolic stability (including CYP3A4 auto-inhibition), and low toxicity risks. Importantly, their ability to act as non-substrates for renal OCT2 transporters suggests potential safety advantages for patients with renal impairment.

In conclusion, the present findings not only identify structurally optimized curcumin derivatives with potent inhibitory effects, especially against SARS-CoV PLpro, but also offer a validated computational framework for future broad-spectrum antiviral drug discovery. The study sets a promising foundation for subsequent in vitro and in vivo investigations and provides valuable insights into designing next-generation antiviral agents capable of countering both current and emerging coronavirus threats.

To facilitate experimental validation, selected compounds such as **10g**, **10d**, and **10c** may be prioritized for chemical synthesis using the proposed synthetic pathways. Their inhibitory effects on PLpro enzymatic activity can be assessed through established biochemical assays, followed by cellular and possibly animal model testing to confirm antiviral efficacy. These steps would provide critical empirical validation of computational predictions and guide the future development of clinically viable antiviral agents.

Nevertheless, several limitations of the current study must be acknowledged. All findings are based on computational models, and no in vitro or in vivo validation has yet been performed. While the ADMET predictions provide early-stage pharmacokinetic insights, experimental confirmation is essential to assess true bioavailability, toxicity, and metabolic stability. Furthermore, synthetic accessibility and potential off-target effects of the designed compounds remain to be investigated.

## Figures and Tables

**Table 1:** Physicochemical and Lipophilicity Properties of Curcumin Derivatives and Their Compliance with Ro5 rules.

	Physicochemical and Liphophilicity Parameters				Drug-Likeness Properties	
Compound Name	MW (Dalton)	HBA	HBD	logP	Violations of Ro5 Rules	Violations
**5a**	472.07	3	1	5.448	1	LOGP > 5.00
**5b**	549.98	3	1	5.815	2	MW > 500, LOGP > 5.00
**5c**	506.03	3	1	5.828	2	MW > 500, LOGP > 5.00
**5d**	490.06	4	1	5.438	1	LOGP > 5.00
**5e**	486.08	3	1	5.758	1	LOGP > 5.00
**5f**	502.08	4	1	5.190	2	MW > 500, LOGP > 5.00
**5g**	488.06	4	2	4.985		
**6a**	428.12	3	1	5.305	1	
**6b**	506.03	3	1	5.781	2	MW > 500, LOGP > 5.00
**6c**	462.08	3	1	5.765	1	LOGP > 5.00
**6d**	446.11	3	1	5.373	1	LOGP > 5.00
**6e**	442.13	3	1	5.724	1	LOGP > 5.00
**6f**	458.13	4	1	5.050	1	LOGP > 5.00
**6g**	444.11	4	2	4.826		
**7a**	412.15	3	1	4.765		
**7b**	490.06	3	1	5.345	1	LOGP > 5.00
**7c**	446.11	3	1	5.332	1	LOGP > 5.00
**7d**	430.14	3	1	4.942		
**7e**	426.16	3	1	5.228	1	LOGP > 5.00
**7f**	442.16	4	1	4.672		
**7g**	428.14	4	2	4.466		
**8a**	408.17	3	1	5.081	1	LOGP > 5.00
**8b**	486.08	3	1	5.669	1	LOGP > 5.00
**8c**	442.13	3	1	5.716	1	LOGP > 5.00
**8d**	426.16	3	1	5.257	1	LOGP > 5.00
**8e**	422.19	3	1	5.519	1	LOGP > 5.00
**8f**	438.18	4	1	5.121	1	LOGP > 5.00
**8g**	424.17	4	2	4.627		
**9a**	424.17	4	1	4.666		
**9b**	502.08	4	1	5.102	2	MW > 500, LOGP > 5.00
**9c**	458.13	4	1	5.038	1	LOGP > 5.00
**9d**	442.16	4	1	4.629		
**9e**	438.18	4	1	4.309		
**9f**	454.18	5	1	4.287		
**9g**	440.16	5	2	4.143		
**10a**	410.15	4	2	4.283		
**10b**	488.06	4	2	4.890		
**10c**	444.11	4	2	4.824		
**10d**	428.14	4	2	4.452		
**10e**	424.17	4	2	4.640		
**10f**	440.16	5	2	4.163		
**10g**	426.15	5	3	3.899		
Curcumin	368.38	6	2	1.47		
Remdesivir	602.58	12	4	0.18	2	MW > 500, HBA > 10
Lopinavir	628.80	5	4	2.93	1	MW > 500

**Table 2:** Predicted Absorption Parameters of Selected Curcumin Derivatives.

Compound	Intestinal Absorption (% Absorbed)	Caco-2 Permeability (log Papp)	P-gp Substrate	P-gp I Inhibitor	P-gp II Inhibitor	Skin Permeability (log Kp)	Water Solubility (log mol/L)
**5g**	93.098	0.515	Yes	Yes	Yes	−2.742	−4.683
**6g**	93.166	0.523	Yes	Yes	Yes	−2.742	−4.669
**7a**	95.753	1.273	Yes	Yes	Yes	−2.734	−5.391
**7d**	96.110	1.296	No	Yes	Yes	−2.735	−4.75
**7f**	97.097	1.258	No	Yes	Yes	−2.735	−4.811
**7g**	94.131	1.049	Yes	Yes	Yes	−2.739	−4.359
**8g**	94.624	0.573	Yes	Yes	Yes	−2.743	−4.629
**9a**	94.310	1.203	Yes	Yes	Yes	−2.736	−6.301
**9d**	94.849	1.259	Yes	Yes	Yes	−2.735	−6.291
**9e**	96.177	1.206	Yes	Yes	Yes	−2.738	−6.232
**9f**	96.108	0.474	Yes	Yes	Yes	−2.734	−6.336
**9g**	92.807	0.505	Yes	Yes	Yes	−2.738	−5.21
**10a**	91.302	0.533	Yes	Yes	Yes	−2.736	−4.492
**10b**	89.716	0.440	Yes	Yes	Yes	−2.737	−5.13
**10c**	89.783	0.448	Yes	Yes	Yes	−2.737	−5.122
**10d**	90.526	0.487	Yes	Yes	Yes	−2.737	−5.043
**10e**	92.590	0.451	Yes	Yes	Yes	−2.741	−5.464
**10f**	91.207	0.514	Yes	Yes	Yes	−2.739	−5.258
**10g**	93.677	0.679	Yes	Yes	Yes	−2.737	−4.048
Curcumin	82.190	−0.093	Yes	Yes	Yes	−2.764	−4.01

**Table 3:** Predicted Distribution Parameters of Selected Curcumin Derivatives.

Compound	VDss (log L/kg)	Fraction Unbound (Fu)	BBB Permeability (log BB)	CNS Permeability (log PS)
**5g**	−0.708	0.000	−0.475	−1.528
**6g**	−0.717	0.000	−0.474	−1.551
**7a**	−0.611	0.038	−0.241	−1.300
**7d**	−0.839	0.073	−0.224	−1.319
**7f**	−0.786	0.042	−0.376	−1.699
**7g**	−0.926	0.044	−0.441	−1.707
**8g**	−0.696	0.000	−0.461	−1.591
**9a**	−0.374	0.032	−0.291	−1.653
**9d**	−0.643	0.040	−0.277	−1.662
**9e**	−0.259	0.035	−0.446	−1.543
**9f**	−0.606	0.031	−0.432	−1.788
**9g**	−0.999	0.000	+0.231	−1.761
**10a**	−0.879	0.000	−0.279	−1.568
**10b**	−0.805	0.000	−0.296	−1.442
**10c**	−0.814	0.000	−0.295	−1.465
**10d**	−0.994	0.000	−0.254	−1.596
**10e**	−0.662	0.000	−0.501	−1.525
**10f**	−0.882	0.000	−0.080	−1.721
**10g**	−1.054	0.000	−0.749	−1.910
Curcumin	−0.215	0.000	−0.562	−2.990

**Table 4:** P450 Enzyme Interactions of Curcumin Derivatives.

Compound	CYP2D6 Substrate	CYP3A4 Substrate	CYP3A4 Inhibitor	CYP1A2 Inhibitor	CYP2C19 Inhibitor	CYP2C9 Inhibitor
**5g**	No	Yes	No	No	Yes	Yes
**6g**	No	Yes	No	No	Yes	Yes
**7a**	No	Yes	No	No	Yes	Yes
**7d**	No	Yes	Yes	Yes	Yes	Yes
**7f**	No	Yes	Yes	No	Yes	Yes
**7g**	No	Yes	Yes	No	Yes	Yes
**8g**	No	Yes	No	No	Yes	Yes
**9a**	No	Yes	No	No	Yes	No
**9d**	No	Yes	No	No	Yes	Yes
**9e**	No	Yes	No	No	Yes	No
**9f**	No	Yes	Yes	No	Yes	Yes
**9g**	No	Yes	Yes	No	Yes	Yes
**10a**	No	Yes	No	No	Yes	Yes
**10b**	No	Yes	No	No	Yes	Yes
**10c**	No	Yes	No	No	Yes	Yes
**10d**	No	Yes	Yes	No	Yes	Yes
**10e**	No	Yes	No	No	Yes	No
**10f**	No	Yes	No	No	Yes	Yes
**10g**	No	Yes	No	No	Yes	Yes
Curcumin	No	Yes	Yes	No	No	No

**Table 5:** Predicted Excretion Parameters of Curcumin Derivatives.

Compound	Total Clearance (log mL/min/kg)	Renal OCT2 Substrate
**5g**	−0.219	No
**6g**	−0.039	No
**7a**	+0.091	No
**7d**	−0.002	No
**7f**	+0.117	No
**7g**	+0.050	No
**8g**	+0.183	No
**9a**	+0.194	No
**9d**	+0.050	No
**9e**	+0.183	No
**9f**	+0.187	No
**9g**	+0.131	No
**10a**	+0.139	No
**10b**	−0.227	No
**10c**	−0.047	No
**10d**	−0.001	No
**10e**	+0.127	No
**10f**	+0.136	No
**10g**	+0.093	No
Curcumin	−0.002	No

**Table 6:** Predicted Toxicological Parameters of Curcumin Derivatives.

Compound	AMES Toxicity	Max. Tolerated Dose (log g/kg/day)	hERG I Inhibitor	Oral Rat Acute Toxicity (LD50, mol/kg)	Oral Rat Chronic Toxicity (LOALEL) (logmg/kgbw/day)	Hepatotoxicity	T. Pyriformis Toxicity (log µg/L)	Minnow Toxicity (log mM)
**5g**	Yes	0.052	No	2.110	1.403	Yes	0.293	−3.283
**6g**	Yes	0.050	No	2.106	1.430	Yes	0.293	−3.137
**7a**	No	0.258	No	2.826	1.404	No	0.296	−2.868
**7d**	No	0.366	No	2.783	2.150	Yes	0.291	−3.714
**7f**	No	0.218	No	2.758	1.964	Yes	0.291	−3.750
**7g**	No	0.178	No	2.016	2.265	Yes	0.290	−3.126
**8g**	Yes	0.047	No	2.077	1.374	Yes	0.293	−2.919
**9a**	No	0.270	No	3.107	1.502	Yes	0.299	−3.088
**9d**	No	0.238	No	2.760	1.254	Yes	0.293	−4.342
**9e**	Yes	0.148	No	3.004	1.423	Yes	0.305	−3.816
**9f**	Yes	0.132	No	2.719	1.045	No	0.293	−4.572
**9g**	Yes	−0.028	No	1.967	1.325	Yes	0.291	−1.909
**10a**	Yes	0.087	No	1.894	1.383	Yes	0.293	−1.273
**10b**	Yes	0.065	No	1.950	1.273	Yes	0.294	−1.832
**10c**	Yes	0.064	No	1.948	1.301	Yes	0.294	−1.686
**10d**	Yes	0.060	No	1.979	1.422	Yes	0.291	−1.341
**10e**	Yes	0.108	No	2.244	1.346	Yes	0.299	−1.005
**10f**	Yes	0.080	No	2.033	1.448	No	0.292	−1.124
**10g**	No	0.089	No	1.853	3.834	No	0.290	−2.312
Curcumin	No	0.081	No	1.833	0.835	No	0.305	−4.572

**Table 7:** Molecular Docking Scores of Curcumin Derivatives, curcuminioids, reference drugs and inhibitors against plpro Enzymes of SARS-CoV, MERS-CoV, and SARS-CoV-2.

	Coranavirus Targets		
Compound Name	6WUU SARS-CoV-2	2FE8 SARS-CoV	4RNA MERS-CoV
**5g**	−9.5	−10.2	−9.0
**6g**	−9.4	−10.1	−9.0
**7a**	−9.3	−10.1	−8.8
**7d**	−9.6	−10.3	−9.1
**7f**	−9.5	−10.3	−9.0
**7g**	−9.7	−10.5	−9.0
**8g**	−9.6	−10.3	−9.1
**9a**	−9.6	−10.3	−9.2
**9d**	−9.8	−10.5	−9.3
**9e**	−9.3	−10.5	−9.1
**9f**	−9.1	−10.5	−9.1
**9g**	−9.6	−10.6	−9.1
**10a**	−9.9	−10.3	−9.2
**10b**	−9.7	−10.4	−9.3
**10c**	−9.8	−10.7	−9.3
**10d**	−10.0	−10.7	−9.4
**10e**	−9.8	−10.6	−9.4
**10f**	−9.8	−10.5	−9.2
**10g**	−10.1	−10.8	−9.3
Bisdemothoxycurcumin	−7.5	−7.5	−7.5
Curcumin	−8.0	−7.8	−7.6
Demothoxycurcumin	−7.9	−8.4	−7.6
Favipiravir	−5.7	−6.0	−5.3
Hydroxychloroquine	−6.7	−6.9	−6.1
Lopinavir	−8.7	−10.1	−8.7
Remdesivir	−8.8	−9.0	−8.1
Warfarin	−8.5	−8.4	−7.4
VIR250	−7.6	−8.2	−6.2

**Table 8:** Binding Free Energy and Their Components of Hit-Scoring Curcumin Derivatives Calculated by MM/PBSA Method.

Residue	Δ*G*^*bind*^	Δ*G*^vW^	Δ*G*^elec^	Δ*G*^ps^	Δ*G*^nps^
**10c**	−259.43	−315.12	−98.65	+148.87	−5.47
**10d**	−268.85	−328.67	−105.23	+157.56	−6.51
**10g**	−280.72	−342.89	−112.75	+165.98	−7.04

**Table 9:** Binding Free Energy Components of Top-Scoring Curcumin Derivatives Calculated by the MM/PBSA Method.

	10c (kJ/mol)		10d (kJ/mol)		10g (kJ/mol)	
Residue	Chain A	Chain C	Chain A	Chain C	Chain A	Chain C
Arg82	1.11	1.75	1.30	2.98	2.45	1.60
Lys108	2.22	1.85	1.51	1.92	1.89	2.35
Asn110	−2.44	−0.32	−2.41	−0.64	−2.27	−0.52
Lys157	1.95	2.32	1.10	2.21	1.45	1.68
Gly161	−2.34	−0.15	−2.11	−0.90	−2.67	−1.56
Glu162	−2.07	−0.22	−1.92	−0.80	−2.45	−0.53
Leu163	−1.98	−1.04	−2.12	−0.98	−2.75	−1.00
Asp164	−2.07	−0.50	−2.45	−0.67	−2.89	−0.10
Glu167	2.75	1.68	2.10	2.20	2.45	3.52
Arg183	3.05	1.85	1.21	3.10	1.75	1.50
Thr198	−0.33	−2.28	−0.23	1.80	−0.30	−2.75
Leu200	−0.25	−2.12	−0.32	−2.35	−0.15	−2.68
Val203	−0.25	−1.97	−0.02	−2.04	−0.11	−2.00
Glu204	1.50	2.34	1.89	1.85	1.22	2.11
Met207	−0.26	−3.78	−0.20	−4.10	−0.50	−4.75
Tyr208	−0.45	−3.10	−0.80	−3.50	−0.05	−3.89
Lys232	1.20	2.05	1.50	2.34	1.85	1.75
Ser245	−2.05	−0.20	−1.78	−0.50	−2.10	−0.85
Pro248	3.75	2.50	1.98	3.75	2.25	4.00
Tyr264	1.10	2.85	1.45	3.15	1.85	3.60
Tyr268	−2.40	−0.10	−0.75	−0.50	−2.00	−0.80
Tyr269	−2.04	−0.02	−1.88	−0.24	−2.07	−0.14
Gln270	−1.98	−0.06	−1.62	−0.19	−2.17	−0.08
Tyr273	−2.05	−0.01	−2.03	−0.20	−1.89	−0.05
Asp302	2.10	2.05	2.50	2.34	2.85	2.70

## Data Availability

Data will be made available upon request.

## Supplementary Materials

The following supporting information can be downloaded at: https://www.mdpi.com/article/10.3390/ph18060798/s1, Scheme S1: Synthetic Route and Rational Design Strategy of Novel Curcumin Derivatives; Table S1: Designed curcumin deriavites; Figure S1: Time evolution of RMSD values for PLpro of SARS-CoV in the protein- hit ligand complex; Figure S2: Predicted ¹H NMR spectrum of compound **10c**; Figure S3: Predicted ¹³C NMR spectrum of compound **10c**; Figure S4: Predicted ¹H NMR spectrum of compound 10d; Figure S5: Predicted ¹³C NMR spectrum of compound 10d; Figure S6: Predicted ¹H NMR spectrum of compound 10g; Figure S7: Predicted ¹³C NMR spectrum of compound **10g**.
