# Supplementary material for: Structure-Based Design and In-Silico Evaluation of Computationally Proposed Curcumin Derivatives as Potential Inhibitors of the Coronaviral PLpro Enzymes
Source: Pharmaceuticals (Basel). 2025 May 26;18(6):798. doi: 10.3390/ph18060798 (PMC12195915; doi:10.3390/ph18060798)
Supplement: Supplementary file 1 [file pharmaceuticals-18-00798-s001.zip › pharmaceuticals-3610672-supplementary.pdf]

Supplementary Materials

Structure-Based Design and In-silico Evaluation of Computationally Proposed Curcumin Derivatives as Potential Inhibitors of the Coronaviral PLpro Enzymes

Hakan Alici

Department of Physics, Faculty of Science, Zonguldak Bülent Ecevit University, 67100 Zonguldak, Türkiye; hakanalici@beun.edu.tr

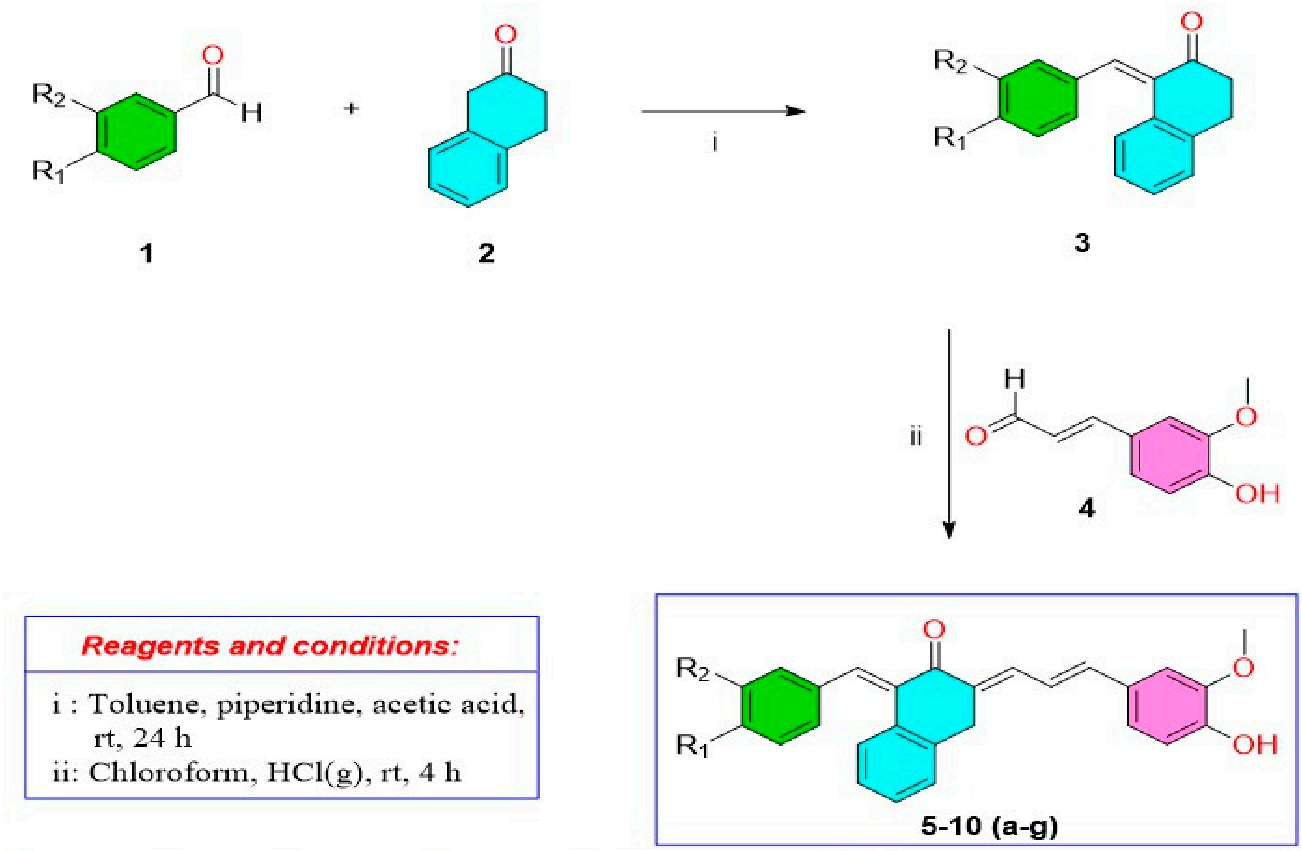

Comp.	R1	R2	Comp.	R1	R2	Comp.	R1	R2
5a	Br	H	7a	F	H	9a	OCH_3	H
5b	Br	Br	7b	F	Br	9b	OCH_3	Br
5c	Br	Cl	7c	F	Cl	9c	OCH_3	Cl
5d	Br	F	7d	F	F	9d	OCH_3	F
5e	Br	CH_3	7e	F	CH_3	9e	OCH_3	CH_3
5f	Br	OCH_3	7f	F	OCH_3	9f	OCH_3	OCH_3
5g	Br	OH	7g	F	OH	9g	OCH_3	OH
6a	Cl	H	8a	CH_3	H	10a	OH	H
6b	Cl	Br	8b	CH_3	Br	10b	OH	Br
6c	Cl	Cl	8c	CH_3	Cl	10c	OH	Cl
6d	Cl	F	8d	CH_3	F	10d	OH	F
6e	Cl	CH_3	8e	CH_3	CH_3	10e	OH	CH_3
6f	Cl	OCH_3	8f	CH_3	OCH_3	10f	OH	OCH_3
6g	Cl	OH	8g	CH_3	OH	10g	OH	OH

Scheme S1. Synthetic Route and Rational Design Strategy of Novel Curcumin Derivatives

- Note: This proposed pathway is not experimentally validated but illustrates how the compounds will be conceptually derived

Table S1: Designed curcumin deriavites

Compound	Name	IUPAC name
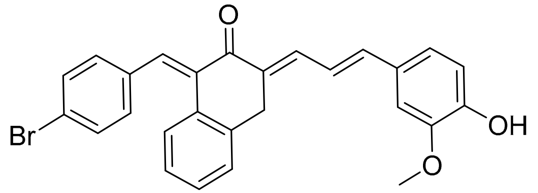	5a	(*E*)-1-((*E*)-4-bromobenzylidene)-3-((*E*)-3-(4-hydroxy-3-methoxyphenyl)allylidene)-3,4-dihydronaphthalen-2(1 *H*)-one
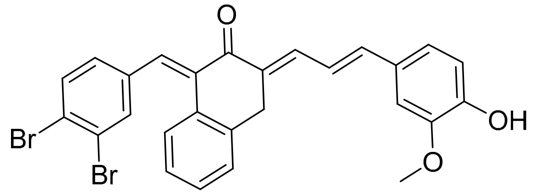	5b	(*E*)-1-((*E*)-3,4-dibromobenzylidene)-3-((*E*)-3-(4-hydroxy-3-methoxyphenyl)allylidene)-3,4-dihydronaphthalen-2(1*H*)-one
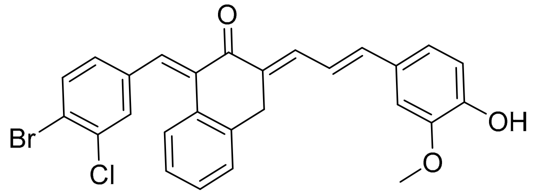	5c	(*E*)-1-((*E*)-4-bromo-3-chlorobenzylidene)-3-((*E*)-3-(4-hydroxy-3-methoxyphenyl)allylidene)-3,4-dihydronaphthalen-2(1*H*)-one
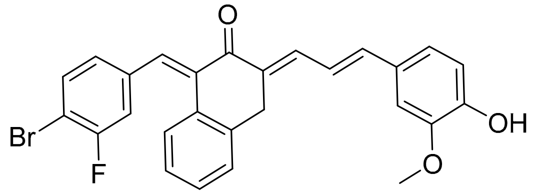	5d	(*E*)-1-((*E*)-4-bromo-3-fluorobenzylidene)-3-((*E*)-3-(4-hydroxy-3-methoxyphenyl)allylidene)-3,4-dihydronaphthalen-2(1*H*)-one
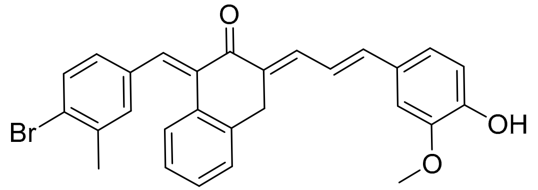	5e	(*E*)-1-((*E*)-4-bromo-3-methylbenzylidene)-3-((*E*)-3-(4-hydroxy-3-methoxyphenyl)allylidene)-3,4-dihydronaphthalen-2(1*H*)-one

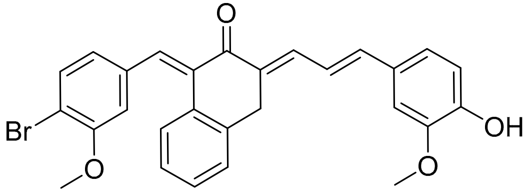	5f	(*E*)-1-((*E*)-4-bromo-3-methoxybenzylidene)-3-((*E*)-3-(4-hydroxy-3-methoxyphenyl)allylidene)-3,4-dihydronaphthalen-2(1*H*)-one
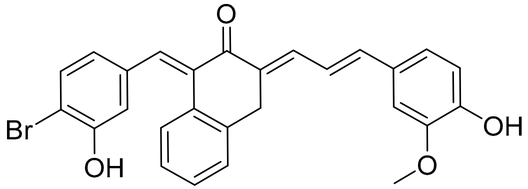	5g	(*E*)-1-((*E*)-4-bromo-3-hydroxybenzylidene)-3-((*E*)-3-(4-hydroxy-3-methoxyphenyl)allylidene)-3,4-dihydronaphthalen-2(1*H*)-one
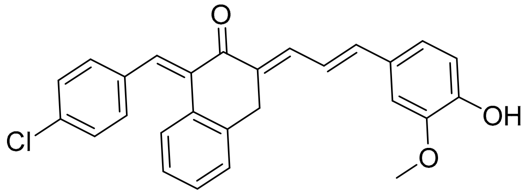	6a	(*E*)-1-((*E*)-4-chlorobenzylidene)-3-((*E*)-3-(4-hydroxy-3-methoxyphenyl)allylidene)-3,4-dihydronaphthalen-2(1 *H*)-one
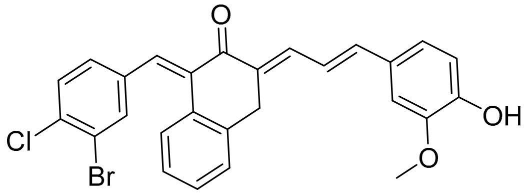	6b	(*E*)-1-((*E*)-3-bromo-4-chlorobenzylidene)-3-((*E*)-3-(4-hydroxy-3-methoxyphenyl)allylidene)-3,4-dihydronaphthalen-2(1*H*)-one
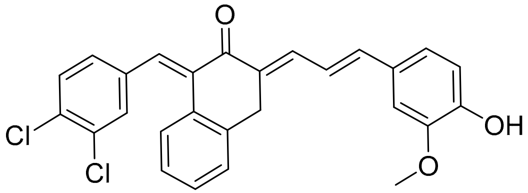	6c	(*E*)-1-((*E*)-3,4-dichlorobenzylidene)-3-((*E*)-3-(4-hydroxy-3-methoxyphenyl)allylidene)-3,4-dihydronaphthalen-2(1*H*)-one
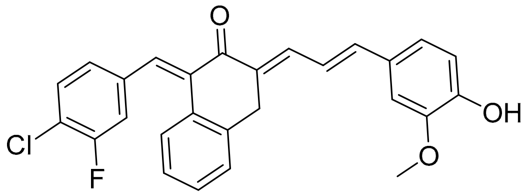	6d	(*E*)-1-((*E*)-4-chloro-3-fluorobenzylidene)-3-((*E*)-3-(4-hydroxy-3-methoxyphenyl)allylidene)-3,4-dihydronaphthalen-2(1*H*)-one

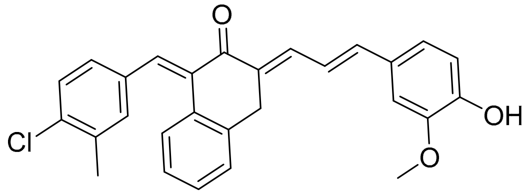	6e	(*E*)-1-((*E*)-4-chloro-3-methylbenzylidene)-3-((*E*)-3-(4-hydroxy-3-methoxyphenyl)allylidene)-3,4-dihydronaphthalen-2(1*H*)-one
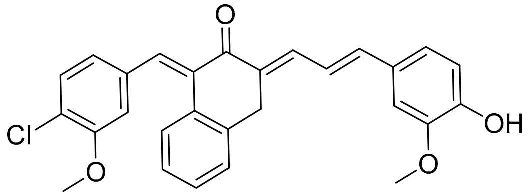	6f	(*E*)-1-((*E*)-4-chloro-3-methoxybenzylidene)-3-((*E*)-3-(4-hydroxy-3-methoxyphenyl)allylidene)-3,4-dihydronaphthalen-2(1*H*)-one
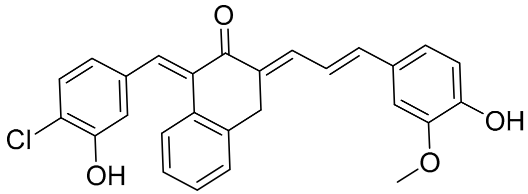	6g	(*E*)-1-((*E*)-4-chloro-3-hydroxybenzylidene)-3-((*E*)-3-(4-hydroxy-3-methoxyphenyl)allylidene)-3,4-dihydronaphthalen-2(1*H*)-one
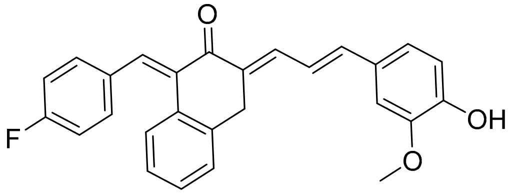	7a	(*E*)-1-((*E*)-4-fluorobenzylidene)-3-((*E*)-3-(4-hydroxy-3-methoxyphenyl)allylidene)-3,4-dihydronaphthalen-2(1*H*)-one
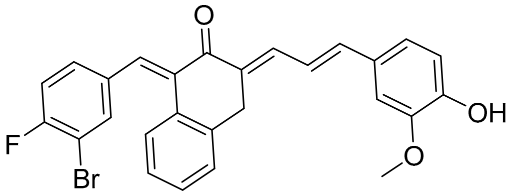	7b	(*E*)-1-((*E*)-3-bromo-4-fluorobenzylidene)-3-((*E*)-3-(4-hydroxy-3-methoxyphenyl)allylidene)-3,4-dihydronaphthalen-2(1*H*)-one

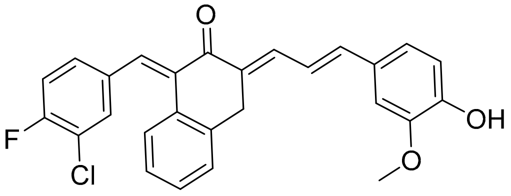 7c	7c	(*E*)-1-((*E*)-3-chloro-4-fluorobenzylidene)-3-((*E*)-3-(4-hydroxy-3-methoxyphenyl)allylidene)-3,4-dihydronaphthalen-2(1*H*)-one
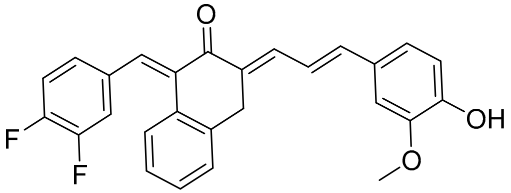	7d	(*E*)-1-((*E*)-3,4-difluorobenzylidene)-3-((*E*)-3-(4-hydroxy-3-methoxyphenyl)allylidene)-3,4-dihydronaphthalen-2(1*H*)-one
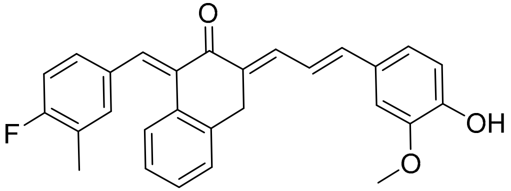	7e	(*E*)-1-((*E*)-4-fluoro-3-methylbenzylidene)-3-((*E*)-3-(4-hydroxy-3-methoxyphenyl)allylidene)-3,4-dihydronaphthalen-2(1 *H*)-one
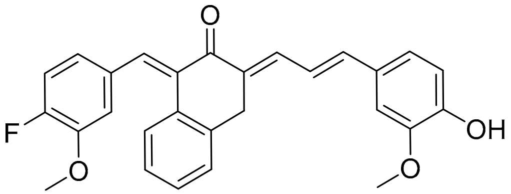	7f	(*E*)-1-((*E*)-4-fluoro-3-methoxybenzylidene)-3-((*E*)-3-(4-hydroxy-3-methoxyphenyl)allylidene)-3,4-dihydronaphthalen-2(1*H*)-one
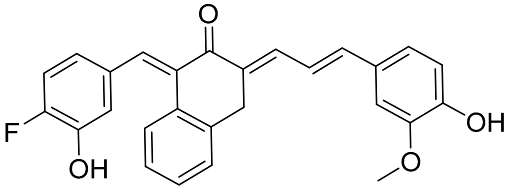	7g	(*E*)-1-((*E*)-4-fluoro-3-hydroxybenzylidene)-3-((*E*)-3-(4-hydroxy-3-methoxyphenyl)allylidene)-3,4-dihydronaphthalen-2(1*H*)-one

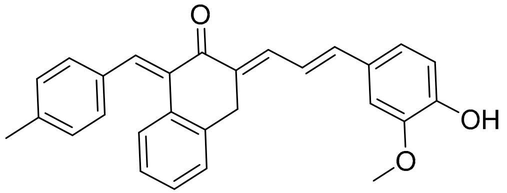	8a	(*E*)-3-((*E*)-3-(4-hydroxy-3-methoxyphenyl)allylidene)-1-((*E*)-4-methylbenzylidene)-3,4-dihydronaphthalen-2(1*H*)-one
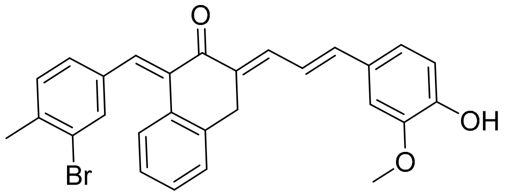	8b	(*E*)-1-((*E*)-3-bromo-4-methylbenzylidene)-3-((*E*)-3-(4-hydroxy-3-methoxyphenyl)allylidene)-3,4-dihydronaphthalen-2(1 *H*)-one
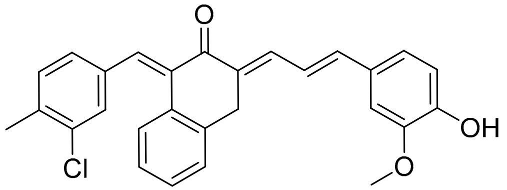	8c	(*E*)-1-((*E*)-3-chloro-4-methylbenzylidene)-3-((*E*)-3-(4-hydroxy-3-methoxyphenyl)allylidene)-3,4-dihydronaphthalen-2(1*H*)-one
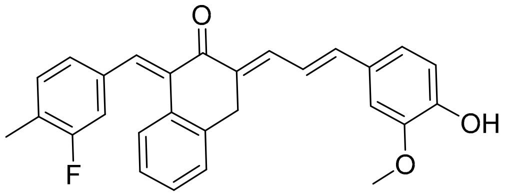	8d	(*E*)-1-((*E*)-3-fluoro-4-methylbenzylidene)-3-((*E*)-3-(4-hydroxy-3-methoxyphenyl)allylidene)-3,4-dihydronaphthalen-2(1 *H*)-one
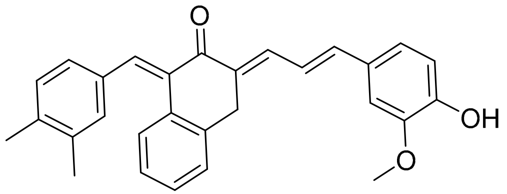	8e	(*E*)-1-((*E*)-3,4-dimethylbenzylidene)-3-((*E*)-3-(4-hydroxy-3-methoxyphenyl)allylidene)-3,4-dihydronaphthalen-2(1*H*)-one
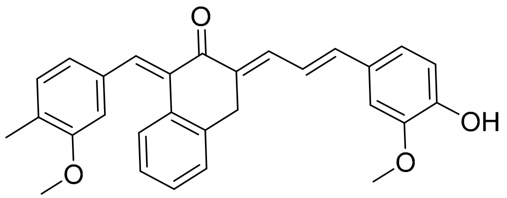	8f	(*E*)-3-((*E*)-3-(4-hydroxy-3-methoxyphenyl)allylidene)-1-((*E*)-3-methoxy-4-methylbenzylidene)-3,4-dihydronaphthalen-2(1*H*)-one

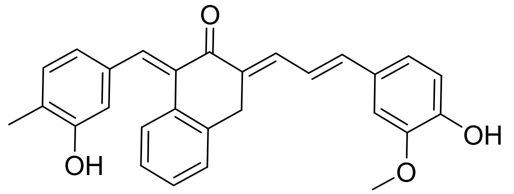	8g	(*E*)-3-((*E*)-3-(4-hydroxy-3-methoxyphenyl)allylidene)-1-((*E*)-3-hydroxy-4-methylbenzylidene)-3,4-dihydronaphthalen-2(1*H*)-one
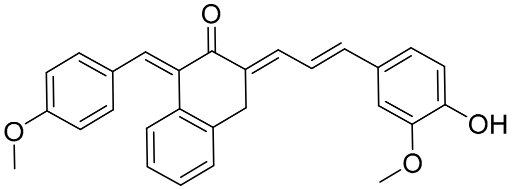	9a	(*E*)-3-((*E*)-3-(4-hydroxy-3-methoxyphenyl)allylidene)-1-((*E*)-4-methoxybenzylidene)-3,4-dihydronaphthalen-2(1*H*)-one
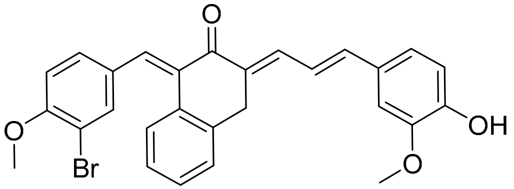	9b	(*E*)-1-((*E*)-3-bromo-4-methoxybenzylidene)-3-((*E*)-3-(4-hydroxy-3-methoxyphenyl)allylidene)-3,4-dihydronaphthalen-2(1*H*)-one
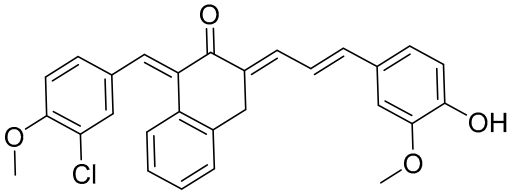	9c	(*E*)-1-((*E*)-3-chloro-4-methoxybenzylidene)-3-((*E*)-3-(4-hydroxy-3-methoxyphenyl)allylidene)-3,4-dihydronaphthalen-2(1*H*)-one
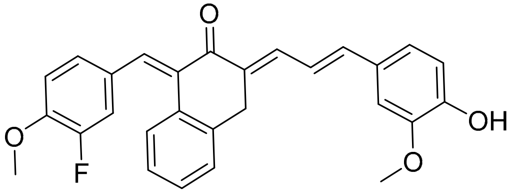	9d	(*E*)-1-((*E*)-3-fluoro-4-methoxybenzylidene)-3-((*E*)-3-(4-hydroxy-3-methoxyphenyl)allylidene)-3,4-dihydronaphthalen-2(1*H*)-one
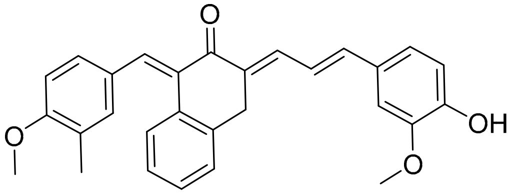	9e	(*E*)-3-((*E*)-3-(4-hydroxy-3-methoxyphenyl)allylidene)-1-((*E*)-4-methoxy-3-methylbenzylidene)-3,4-dihydronaphthalen-2(1*H*)-one

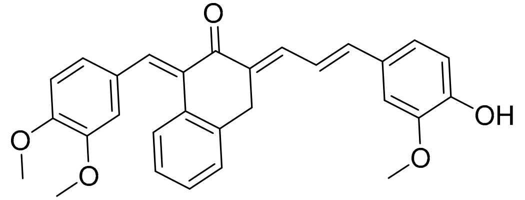	9f	(*E*)-1-((*E*)-3,4-dimethoxybenzylidene)-3-((*E*)-3-(4-hydroxy-3-methoxyphenyl)allylidene)-3,4-dihydronaphthalen-2(1*H*)-one
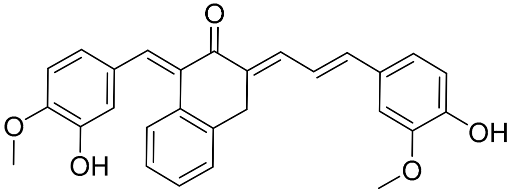	9g	(*E*)-3-((*E*)-3-(4-hydroxy-3-methoxyphenyl)allylidene)-1-((*E*)-3-hydroxy-4-methoxybenzylidene)-3,4-dihydronaphthalen-2(1*H*)-one
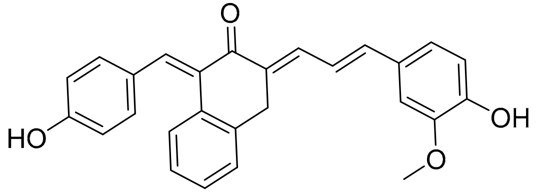	10a	(*E*)-3-((*E*)-3-(4-hydroxy-3-methoxyphenyl)allylidene)-1-((*E*)-4-hydroxybenzylidene)-3,4-dihydronaphthalen-2(1*H*)-one
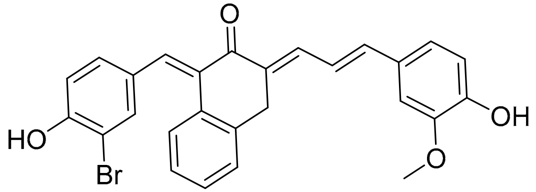	10b	(*E*)-1-((*E*)-3-bromo-4-hydroxybenzylidene)-3-((*E*)-3-(4-hydroxy-3-methoxyphenyl)allylidene)-3,4-dihydronaphthalen-2(1*H*)-one
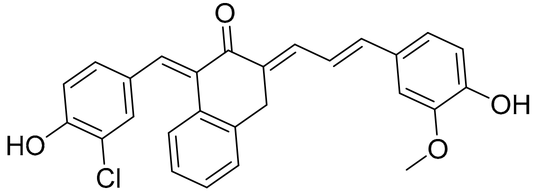	10c	(*E*)-1-((*E*)-3-chloro-4-hydroxybenzylidene)-3-((*E*)-3-(4-hydroxy-3-methoxyphenyl)allylidene)-3,4-dihydronaphthalen-2(1*H*)-one
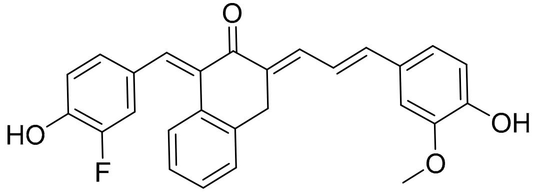	10d	(*E*)-1-((*E*)-3-fluoro-4-hydroxybenzylidene)-3-((*E*)-3-(4-hydroxy-3-methoxyphenyl)allylidene)-3,4-dihydronaphthalen-2(1*H*)-one

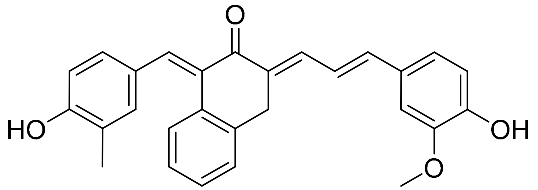	10e	(*E*)-3-((*E*)-3-(4-hydroxy-3-methoxyphenyl)allylidene)-1-((*E*)-4-hydroxy-3-methylbenzylidene)-3,4-dihydronaphthalen-2(1*H*)-one
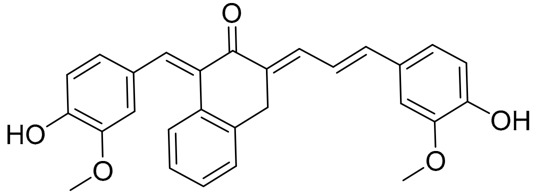	10f	(*E*)-1-((*E*)-4-hydroxy-3-methoxybenzylidene)-3-((*E*)-3-(4-hydroxy-3-methoxyphenyl)allylidene)-3,4-dihydronaphthalen-2(1*H*)-one
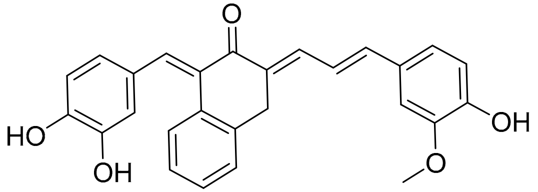	10g	(*E*)-1-((*E*)-3,4-dihydroxybenzylidene)-3-((*E*)-3-(4-hydroxy-3-methoxyphenyl)allylidene)-3,4-dihydronaphthalen-2(1*H*)-one

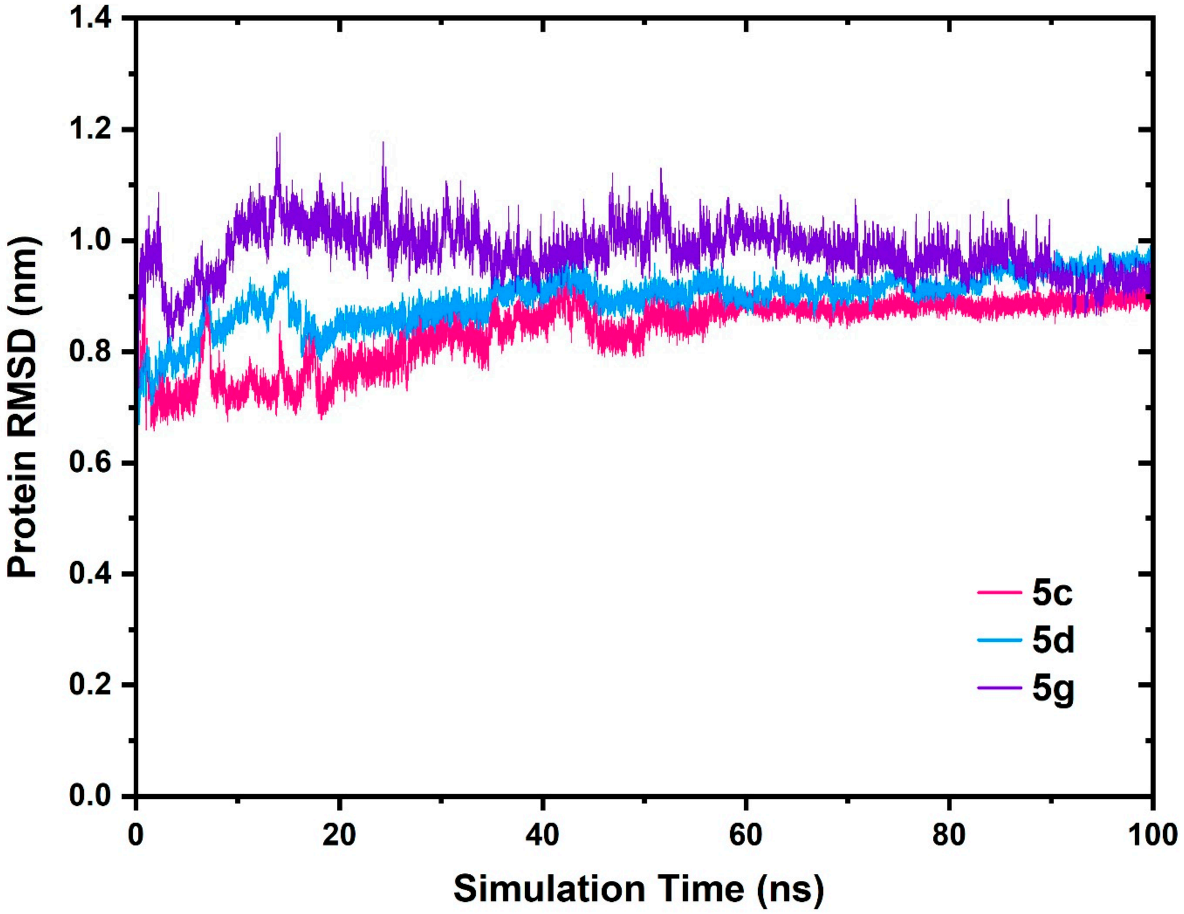

Figure S1. Time evolution of RMSD values for PLpro of SARS-CoV in the protein- hit ligand complex.

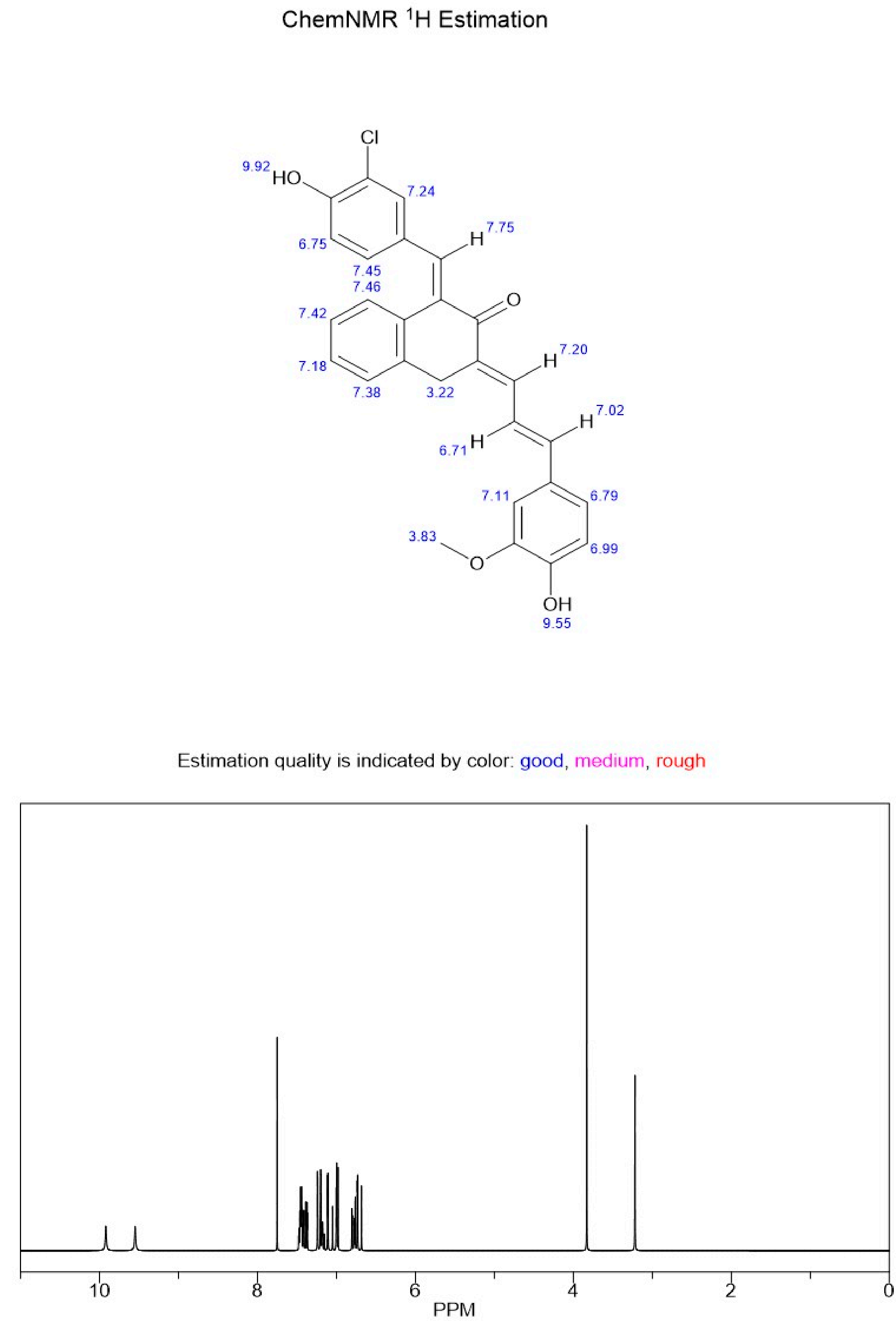

Figure S2. Predicted ^{1}H NMR spectrum of compound 10c.

ChemNMR ^{13}C Estimation

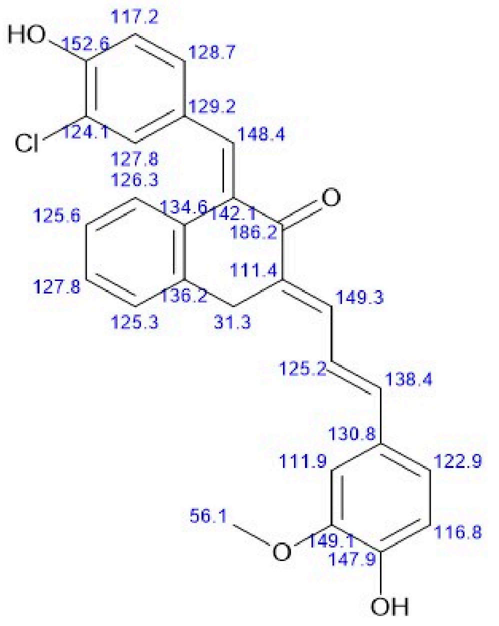

Estimation quality is indicated by color: good, medium, rough

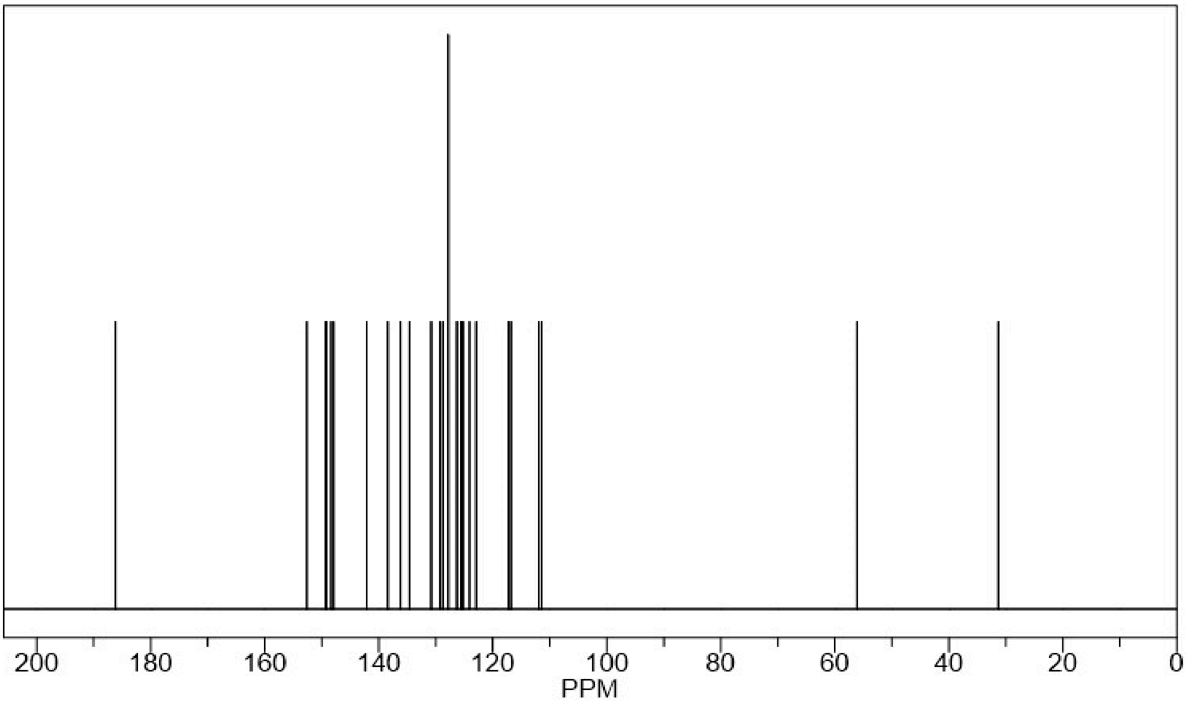

Figure S3. Predicted ^{13}C NMR spectrum of compound 10c.

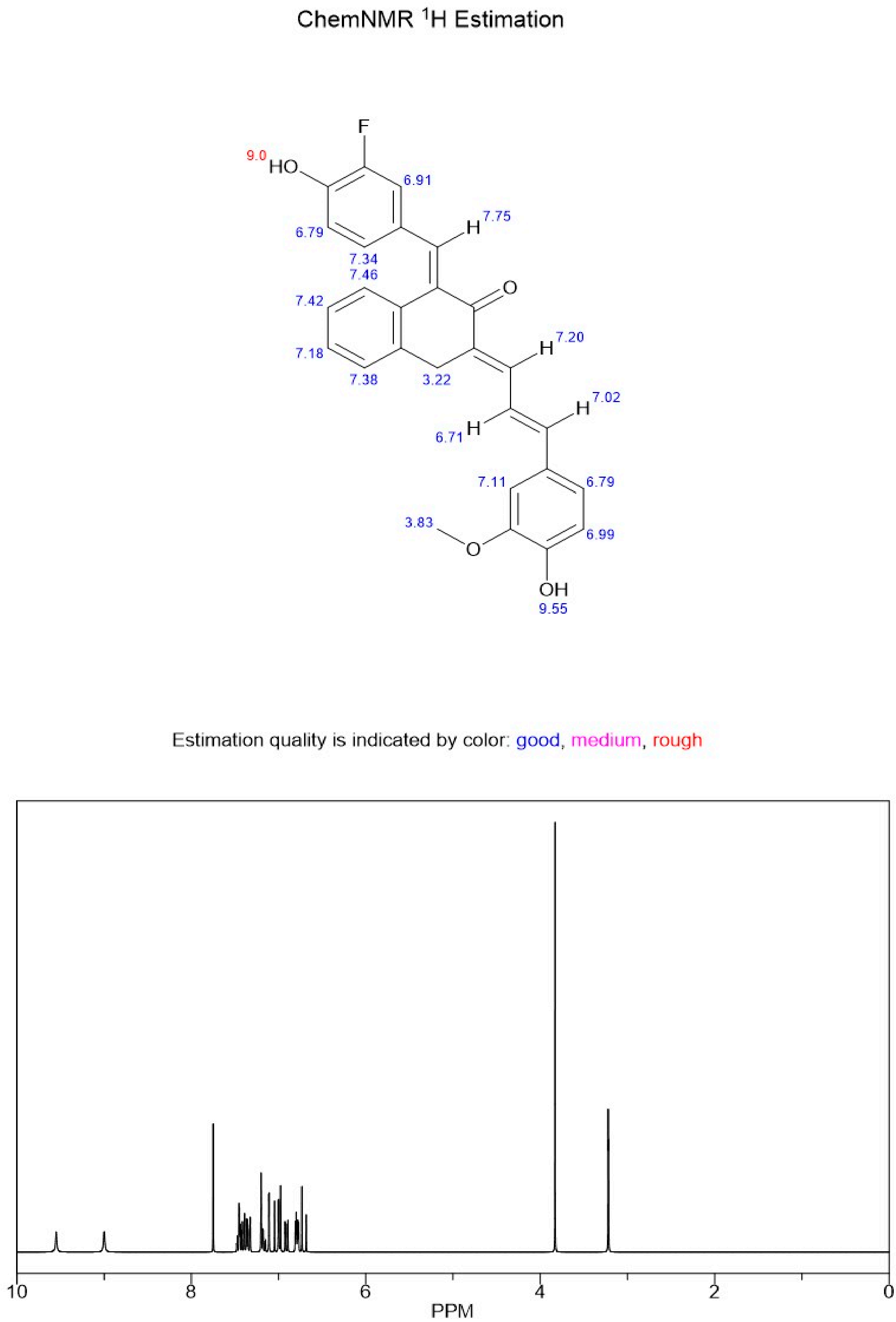

Figure S4. Predicted ^{1}H NMR spectrum of compound 10d.

ChemNMR ^{13}C Estimation

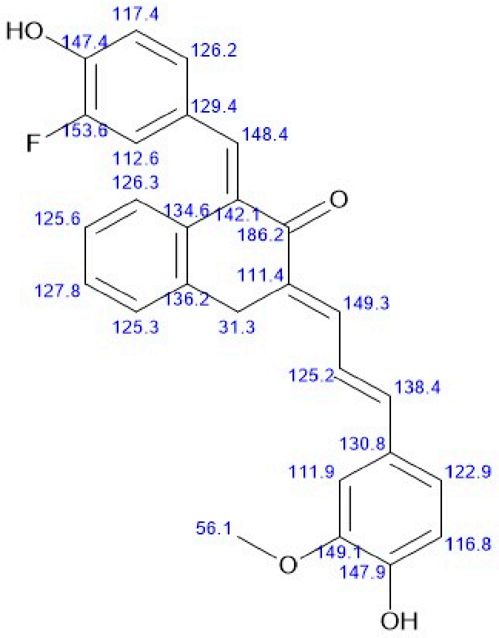

Estimation quality is indicated by color: good, medium, rough

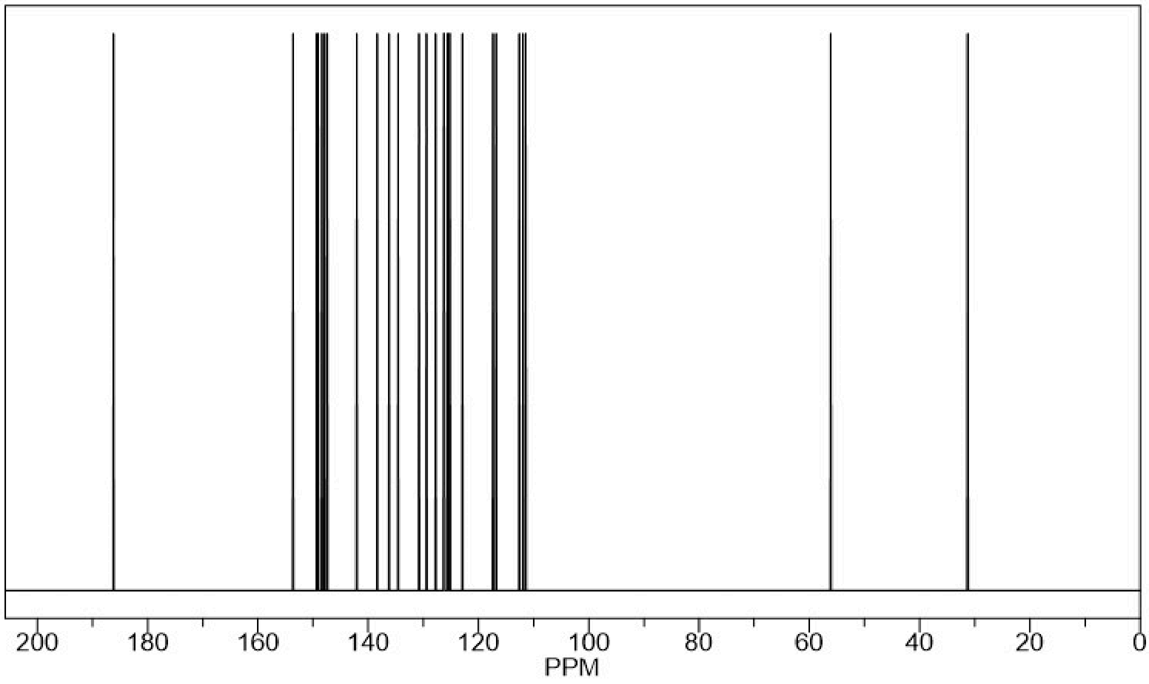

Figure S5. Predicted ^{13}C NMR spectrum of compound 10d.

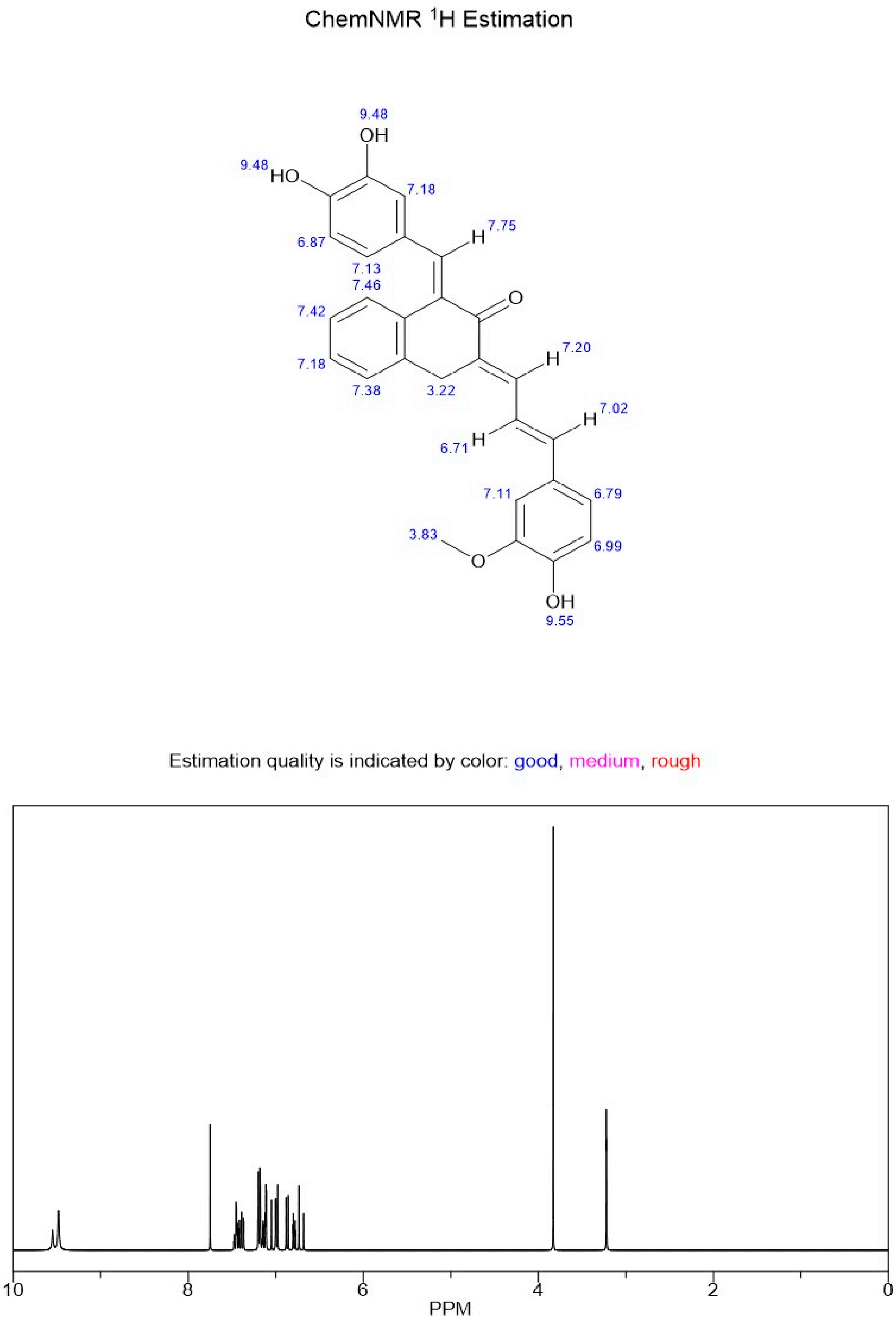

Figure S6. Predicted ^{1}H NMR spectrum of compound 10g

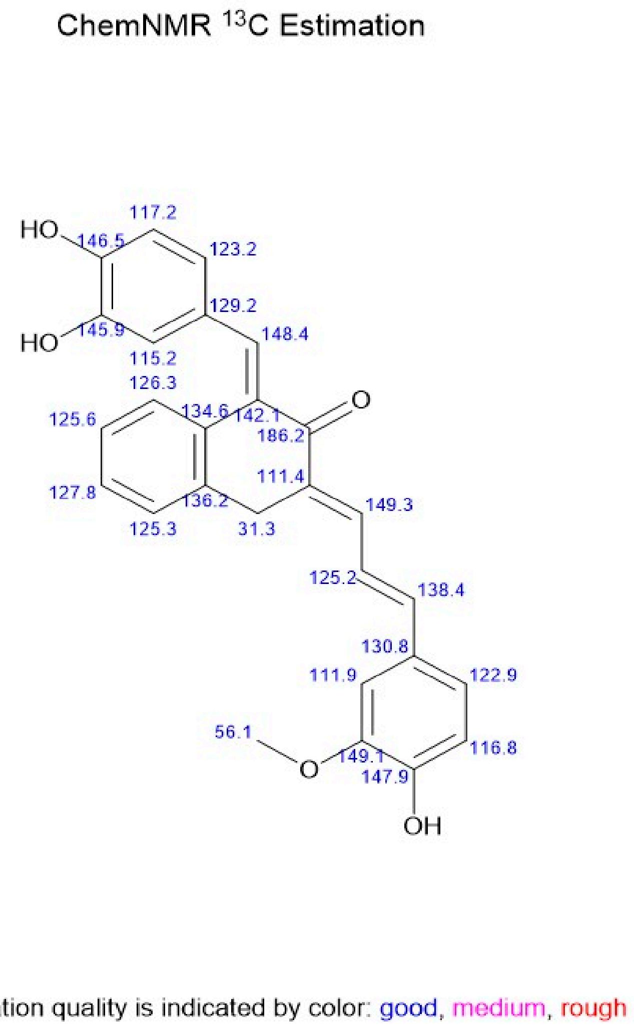

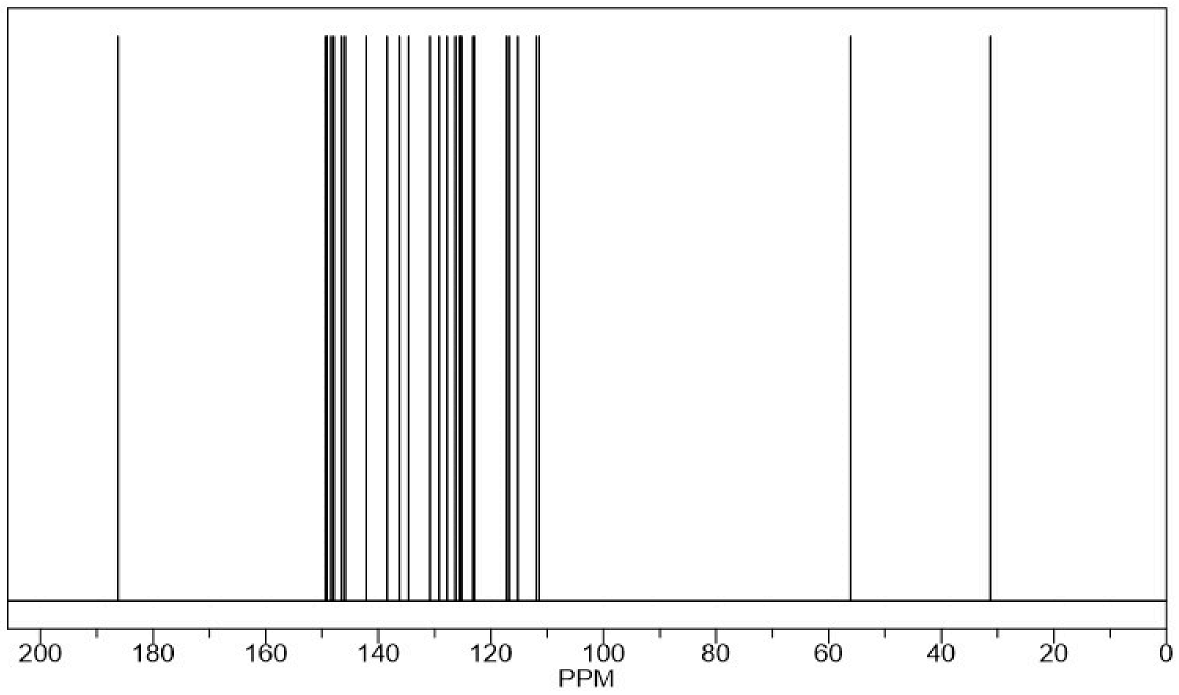

Figure S7. Predicted ^{13}C NMR spectrum of compound 10g
